# Supplementary material for: Intuitive physical reasoning about objects’ masses transfers to a visuomotor decision task consistent with Newtonian physics
Source: PLoS Comput Biol. 2020 Oct 19;16(10):e1007730. doi: 10.1371/journal.pcbi.1007730 (PMC7647116; doi:10.1371/journal.pcbi.1007730)
Supplement: S1 Appendix — (PDF) [file pcbi.1007730.s001.pdf]

Supporting information: Intuitive physical reasoning about objects' masses transfers to a visuomotor decision task consistent with Newtonian physics

Nils Neupärtl[1,2*], Fabian Tatai[1,2], Constantin A. Rothkopf[1,2,3]

1 Centre for Cognitive Science, Technical University of Darmstadt, 64283 Darmstadt, Germany
2 Institute of Psychology, Technical University of Darmstadt, 64283 Darmstadt, Germany
3 Frankfurt Institute for Advanced Studies, Goethe University, 60438 Frankfurt, Germany

* neupaertl@psychologie.tu-darmstadt.de

Puck Motion

From Newtonian physics we know the relationships between a change in momentum Δp by a force F exerted over a time Δt:

$$\Delta p = \mathrm{F}\,\Delta t \tag{1}$$

The impulse is transferred to a puck of mass m resulting in a change of speed Δv:

$$\Delta p = m\,\Delta v \tag{2}$$

As the puck is initially at rest, the release velocity v_0 when shooting the puck can therefore be expressed as:

$$v_0 = \frac{\mathrm{F}\,\Delta t}{m} \propto \Delta t \tag{3}$$

Therefore, in the simulations the change in momentum Δp increases linearly with press-time Δt and proportionally to force F and thus the initial velocity v_0 also scales linearly with the press-time. Once released, a frictional force F_{fr}, which can be expressed in terms of the gravitational force F_g and the friction coefficient μ:

$$F_{fr} = \mu F_g = \mu m g, \tag{4}$$

which slows the puck down with an acceleration a_{fr}, which accordingly to Newton's second law $F = ma$ is:

$$a_{fr} = \mu g \tag{5}$$

until at rest after some time T:

$$v_T = 0 = v_0 - a_{fr} T \tag{6}$$

During this time the puck has moved a distance s_T

$$s_T = \frac{1}{2} a_{fr} T^2 \tag{7}$$

Solving eq. 6 for T and substituting into eq. 7, substituting the acceleration a_{fr} from eq. 5 and using the expression for the initial velocity v_0 from eq. 3 allows finding the press-time required for propelling the puck over a distance Δx in manuscript eq. 1.

Position and velocity updates per frame:

We used the difference equations corresponding to the above equations of motion:

$$x_{t+\delta t} = x_t + v_t \delta t \tag{8}$$

$$v_{t+\delta t} = v_t - a_{fr} \delta t. \tag{9}$$
