# Supplementary figures and images for: Intuitive physical reasoning about objects’ masses transfers to a visuomotor decision task consistent with Newtonian physics
Source: PLoS Comput Biol. 2020 Oct 19;16(10):e1007730. doi: 10.1371/journal.pcbi.1007730 (PMC7647116; doi:10.1371/journal.pcbi.1007730)

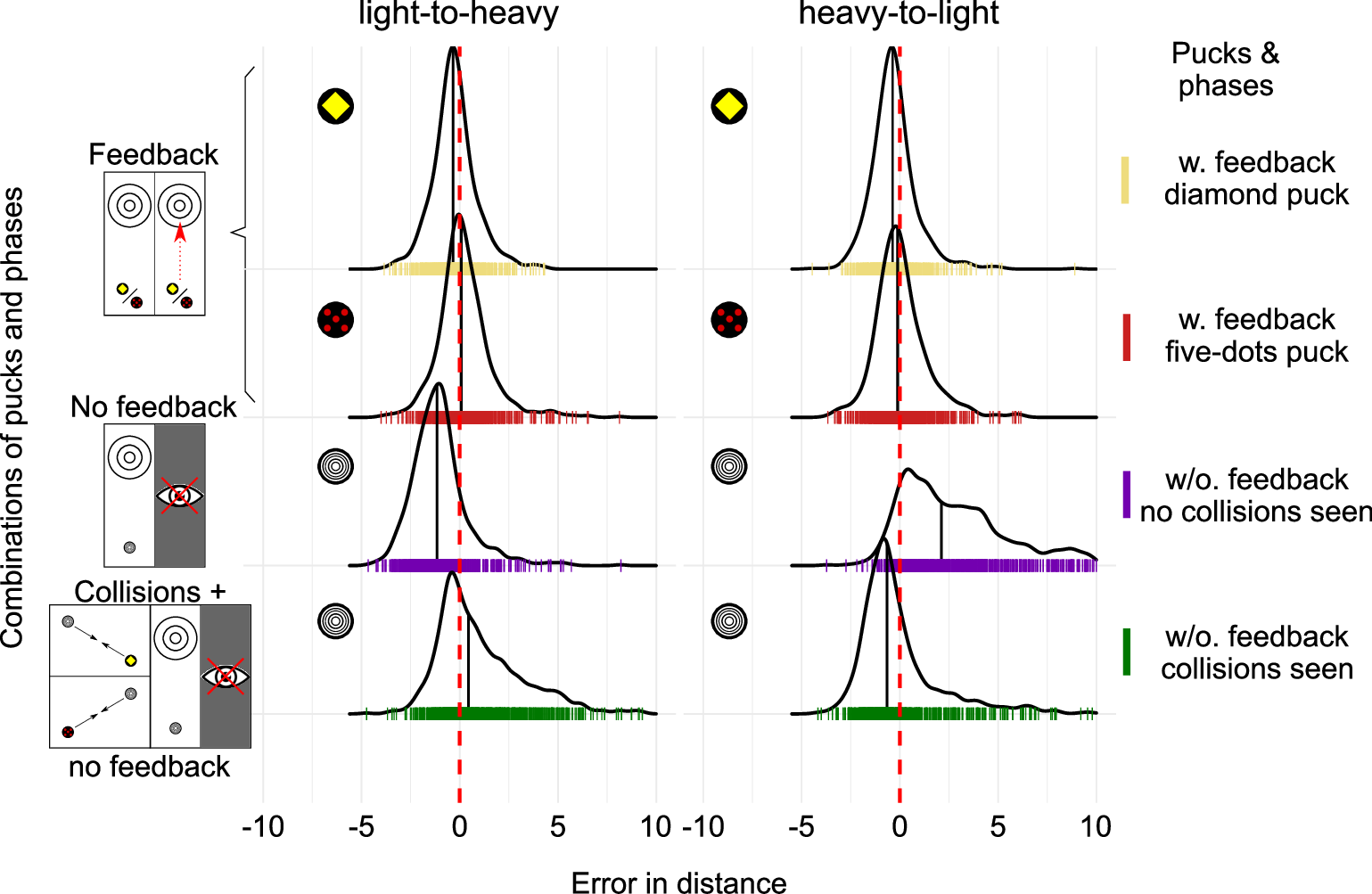

Supplement: S1 Fig — Final discrepancy between target and puck pooled for all participants. Pucks being shot too short are shown with negative values, pucks with a positive deviation were shot too far. Columns showing the the data for both conditions and rows divide into puck and phase combinations. The first two rows (in gold and red) showing the error distributions for both pucks with feedback in phase 2. The error distribution for the unknown puck in phase 3 before seeing the collisions is shown in the second last row (in purple) with greater deviation, with a clear bias and bigger spread. In the last row the error distributions are depicted for the unknown puck after having seen the collisions with the previous learned pucks, showing a reduced bias. (TIF) [file pcbi.1007730.s002.tif]

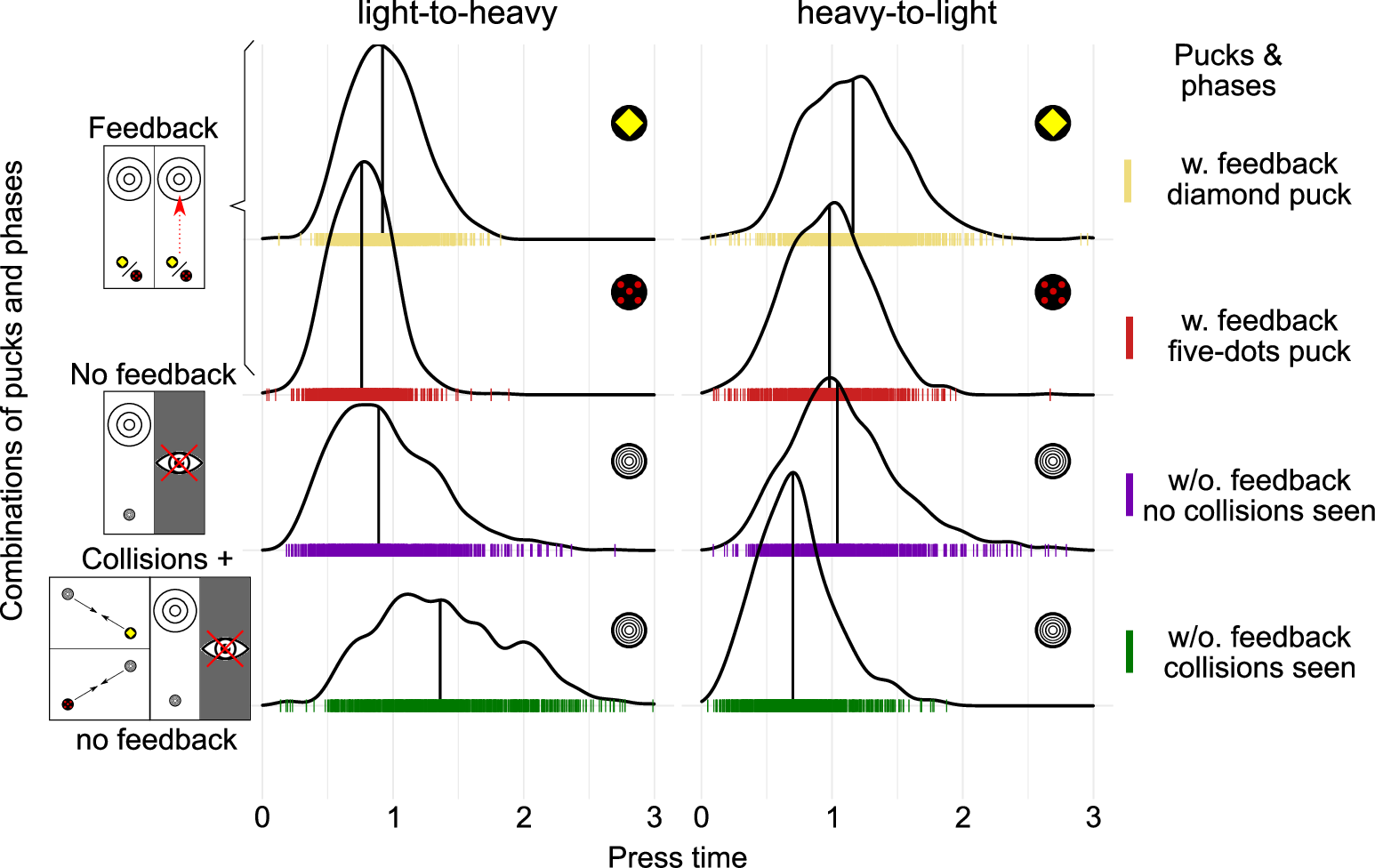

Supplement: S2 Fig — Pooled press-time distributions for all participants. Columns showing the the data for both conditions and rows divide into puck and phase combinations. First two rows showing the press-times for the pucks with feedback. Press-time distributions in phase 3 without feedback are shown in row three in blue. Without further information participants’ behavior in phase 3 is strongly influenced by the previous phase and its press-time distribution: press-time distributions for the unknown puck in phase 3 reflect roughly the combined distributions of press-times of the previous pucks in phase 2 (Kolmogorov D = 0.0538; p = 0.092 for heavy-to-light, D = 0.156; p = 9.8 × 10^−12^ for light-to-heavy). (TIF) [file pcbi.1007730.s003.tif]

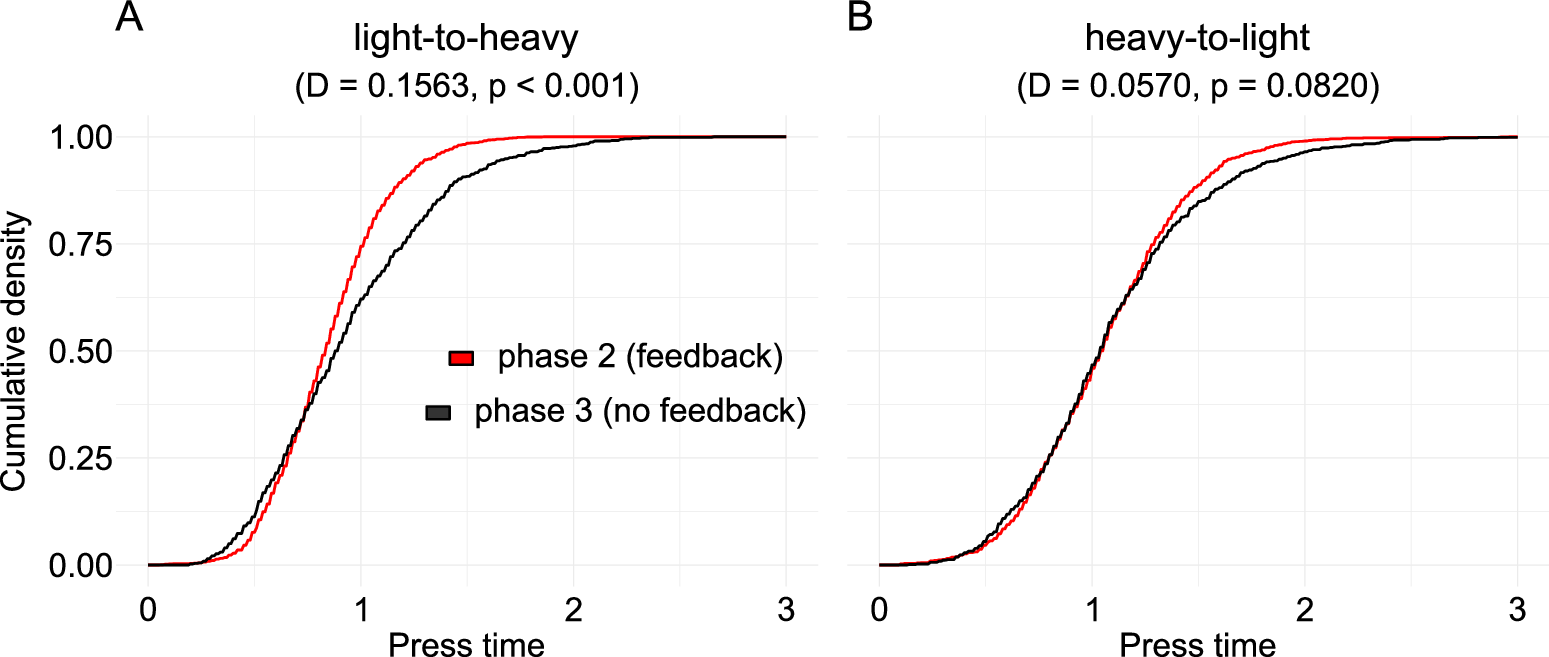

Supplement: S3 Fig — In the light-to-heavy condition both distributions of press times when seeing pucks and without feedback in phase 3 differ significantly. However, considering the asymmetry within the task response—press-times and potential masses are only constrained single-sided towards lower values with a minimum at zero—this difference in press-time distributions is surprisingly small. (B) In the heavy-to-light condition there was no significant difference between the distribution of press-times of both combined feedback pucks and the unknown puck before observing the collisions as revealed by the Kolmogorov-Smirnov test. This suggests that participants adhere to their previous adjusted strategies when facing decisions in great uncertainty. (TIF) [file pcbi.1007730.s004.tif]

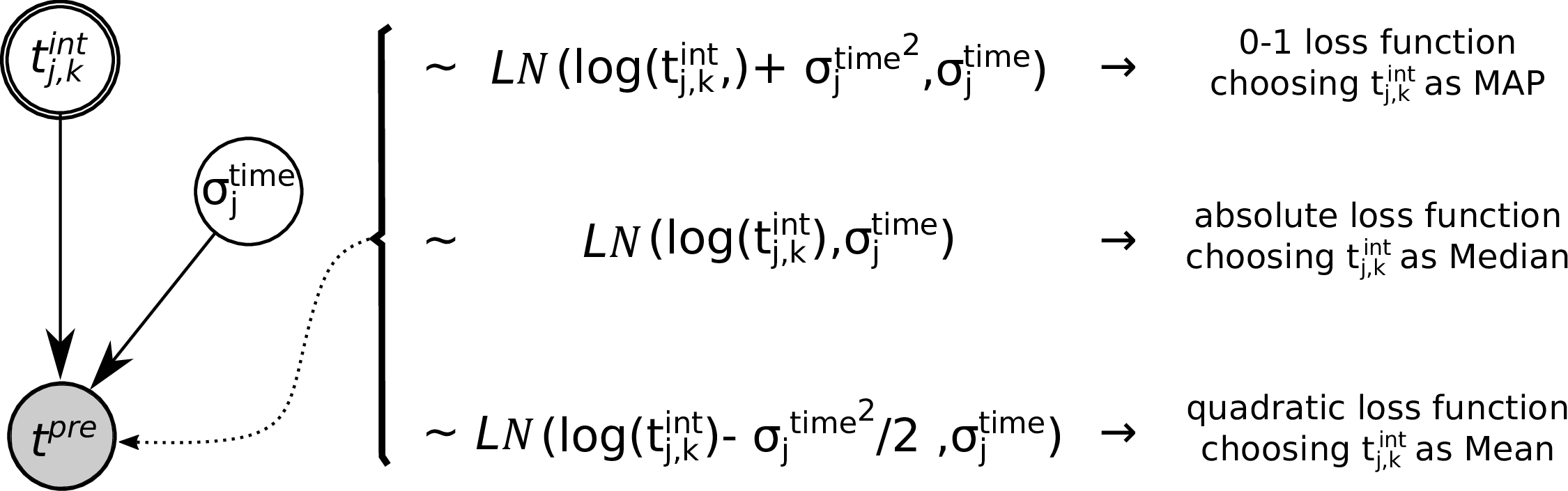

Supplement: S4 Fig — Derivation of the three cost function models based on the expressions for the measures of the central tendency of the log-normal distribution with its mode exp(μ − σ2), median exp(μ) and mean exp(μ+σ22). Setting the intended press-time to one of these measures for the press-time distribution is equivalent with choosing the 0-1, absolute or quadratic loss function. Transformation with the intended press-time tint leads to expressions in S4 Fig. (TIF) [file pcbi.1007730.s005.tif]

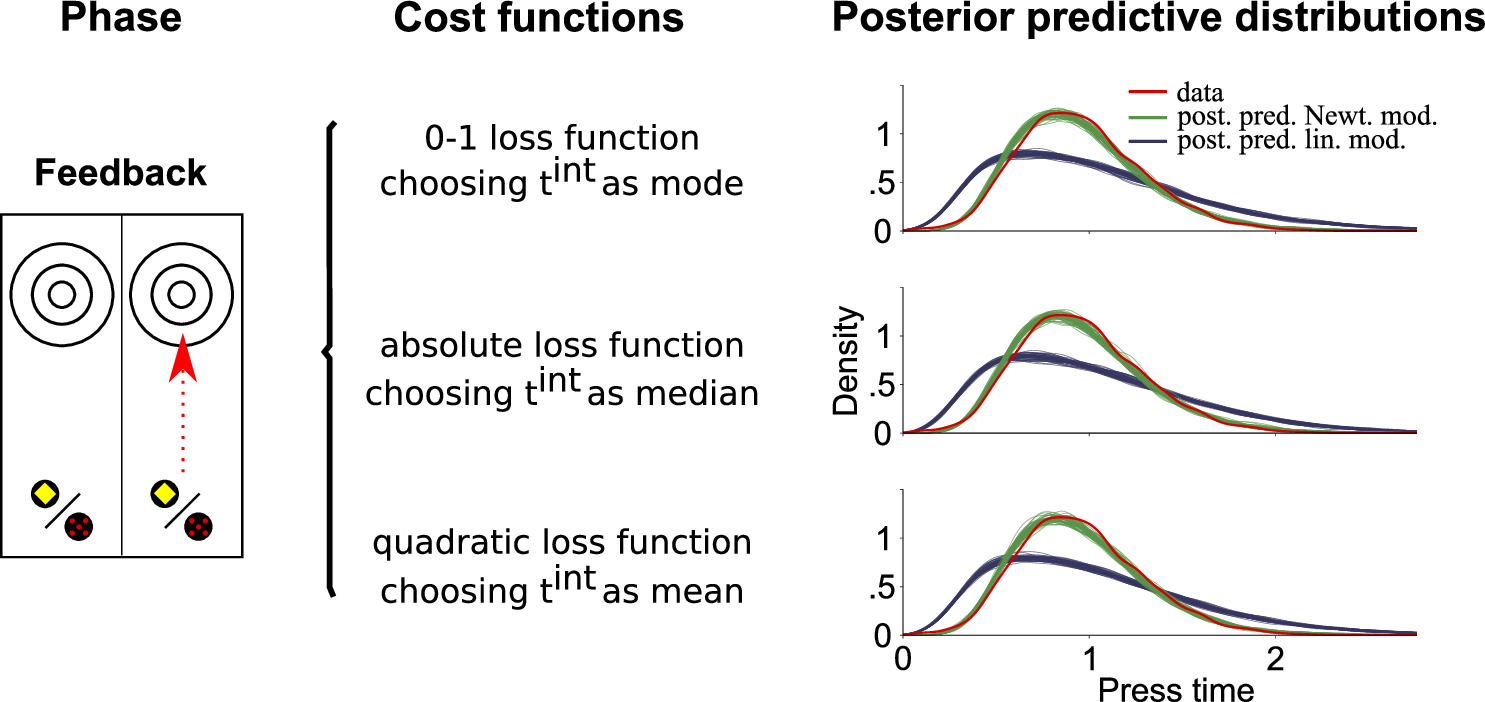

Supplement: S5 Fig — Posterior predictive distributions for both model classes and all cost functions with data from phase 2 with feedback. Posterior predictive distributions of press-times given data from feedback trials. Fifty distributions were drawn from each model after being fitted to the data. Dark green distributions arise from models of the Newtonian model class, dark blue ones from the linear model class. Separation into rows is made on basis of the implemented cost function. For each cost function the Newtonian model predicts values that match the actual data shown as red curve obviously better than the model from the linear model class. (TIF) [file pcbi.1007730.s006.tif]

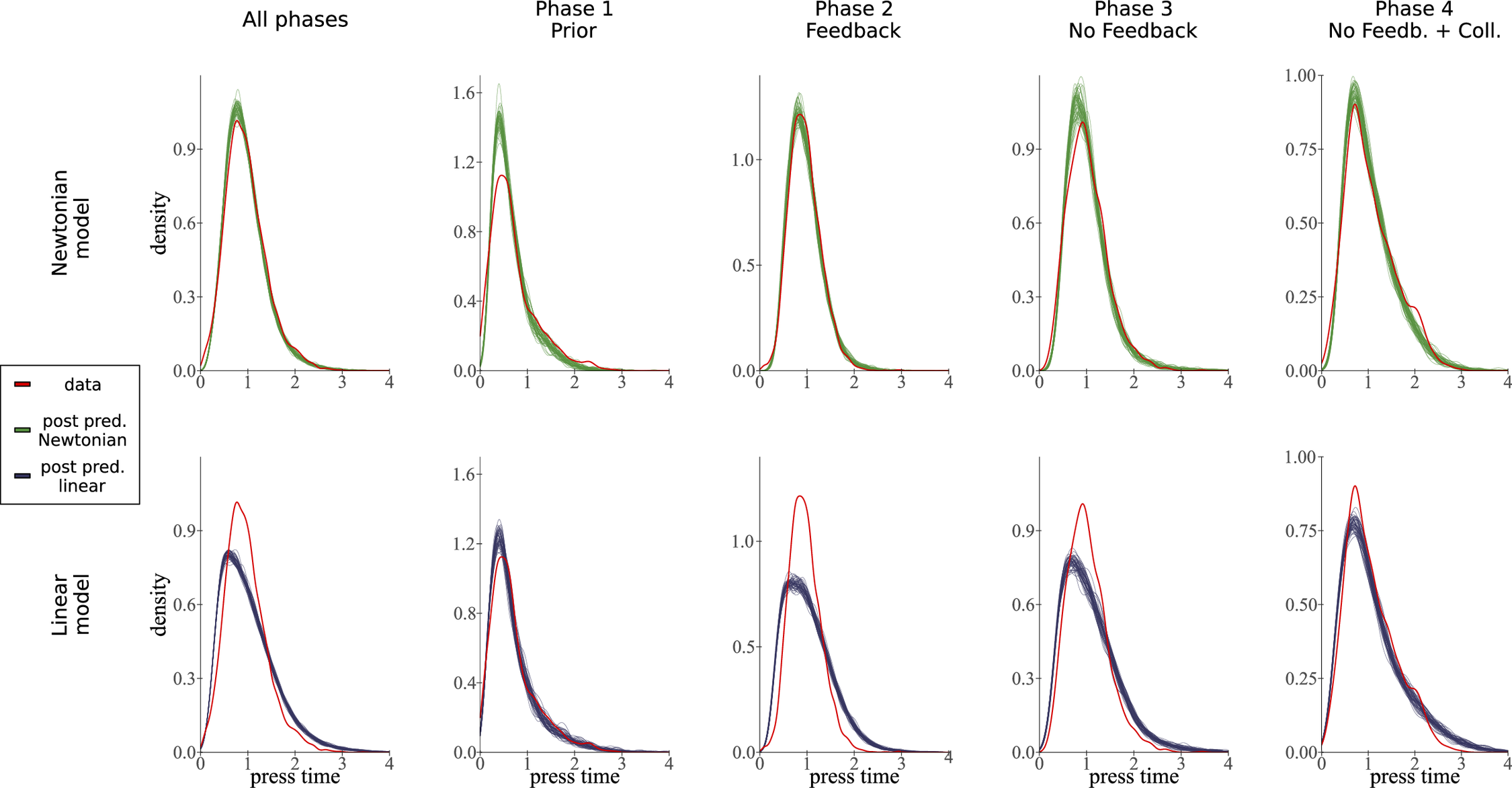

Supplement: S6 Fig — Posterior press-time predictions for both, the linear and the Newtonian model with quadratic cost function, and separately for every phase. Actual data is shown as red line. Model predictions in dark green (50 iterations) of the fitted Newtonian model match the data closely and surpass the fitted linear model in dark blue for the complete data set and in almost every phase individually. (TIF) [file pcbi.1007730.s007.tif]

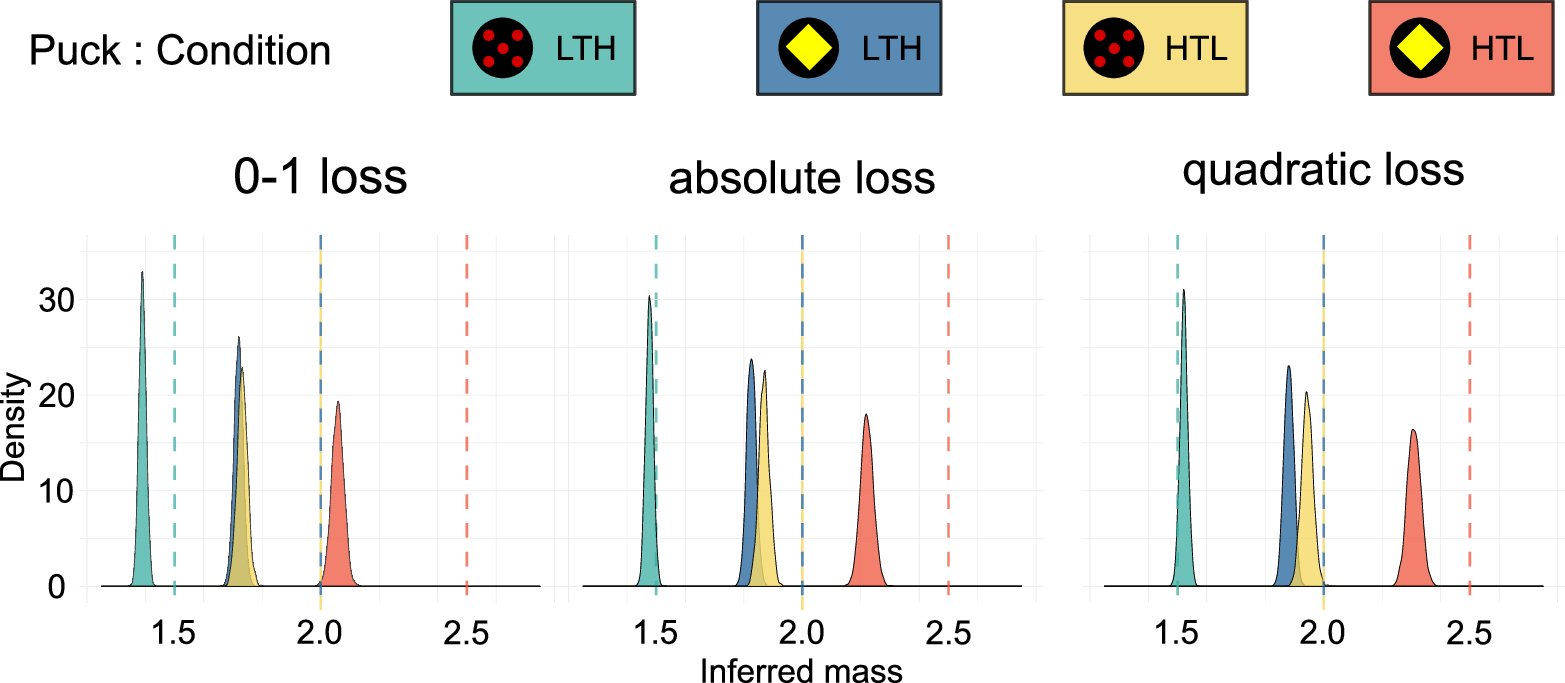

Supplement: S7 Fig — Inferred latent mass beliefs with aggregated data from phase ‘feedback’ for each cost function. Posterior distributions for mass belief aggregated over all participants for each cost function. Colored, vertical lines indicate actual mass of pucks. In comparison the quadratic loss function leads to posterior distributions that fit closest to the actual masses in the experiment. (TIF) [file pcbi.1007730.s008.tif]

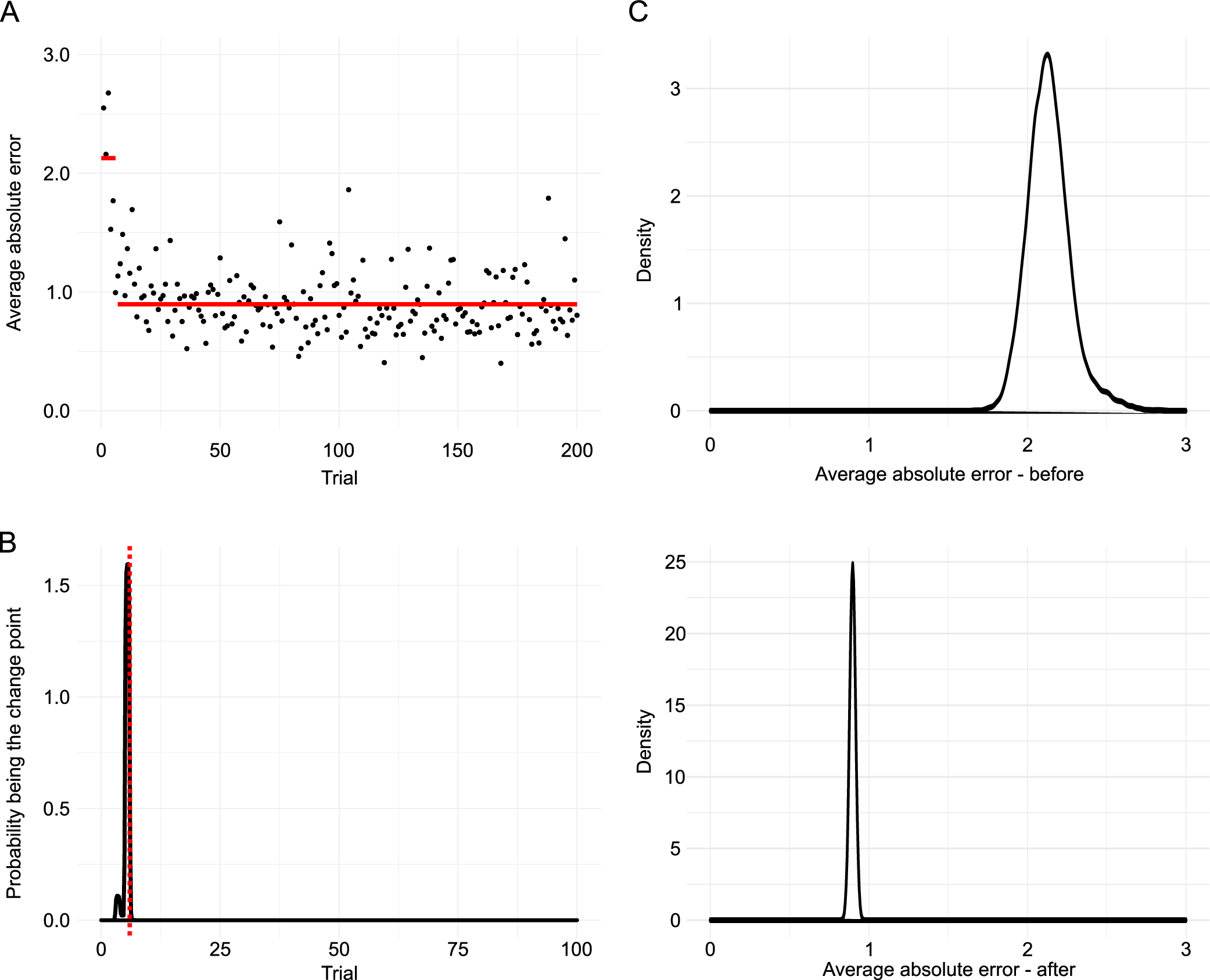

Supplement: S8 Fig — Average absolute error as function of trials and posterior of mean average error derived using the change point detection model. (A) Average absolute error over participants as function of trial number. (B) Posterior over change point τ. Red dotted line marks trial six. (C) Posterior of mean error before and after change point. (TIF) [file pcbi.1007730.s009.tif]

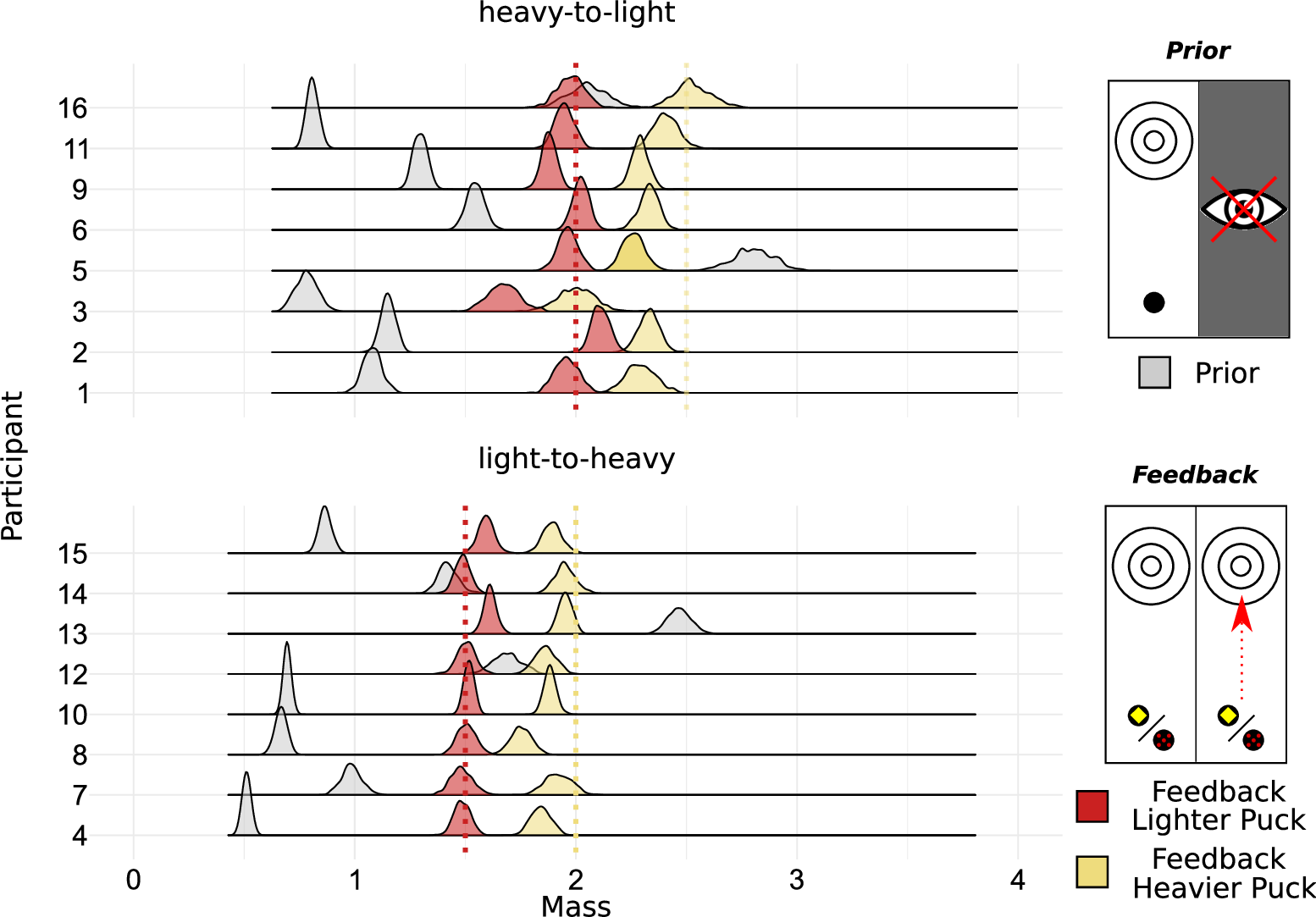

Supplement: S9 Fig — Inferred latent mass in Newtonian model class with quadratic loss function for each participant and with data from Prior and Feedback phase. Posterior mass distributions for each participant in Prior and Feedback phase. Gray distributions show the inferred mass distribution for an unknown puck before participants have encountered the task dynamics. Resulting mass distributions for both pucks in feedback trials in red (light puck) and yellow (heavy puck). Dotted lines indicate actually implemented mass for each of the feedback pucks. (TIF) [file pcbi.1007730.s010.tif]

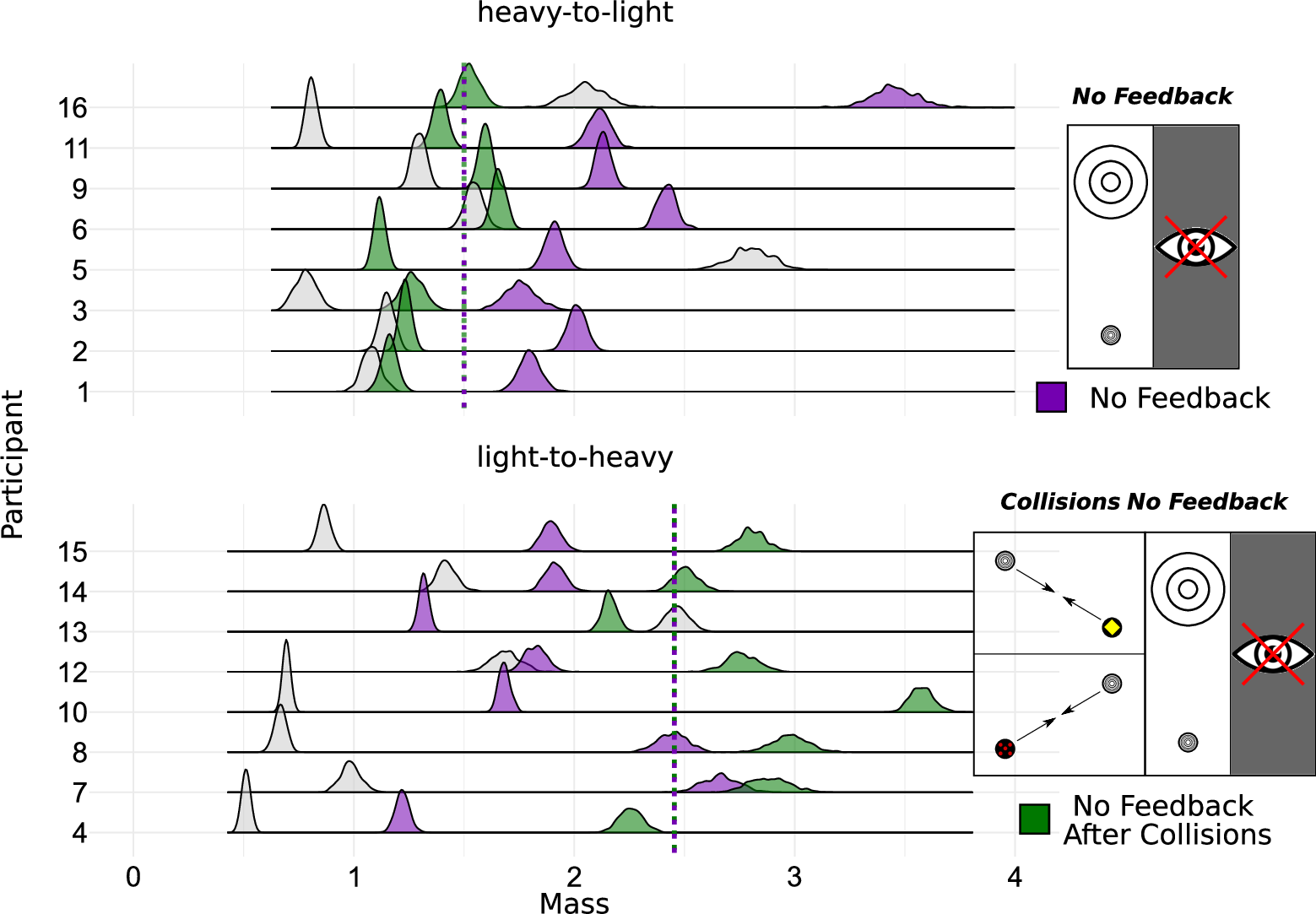

Supplement: S10 Fig — Inferred latent mass in Newtonian model class with quadratic loss function for each participant with data from Prior and both No Feedback phases. Posterior mass distributions for each participant in Prior and Feedback phase. Gray distributions show again the inferred mass distribution for an unknown puck before participants have encountered the task dynamics. Distributions in violet and green are the posterior mass distributions of the unknown puck without feedback before and after the participants saw collision with known pucks. Dotted line marks the actual mass of the unknown puck. (TIF) [file pcbi.1007730.s011.tif]

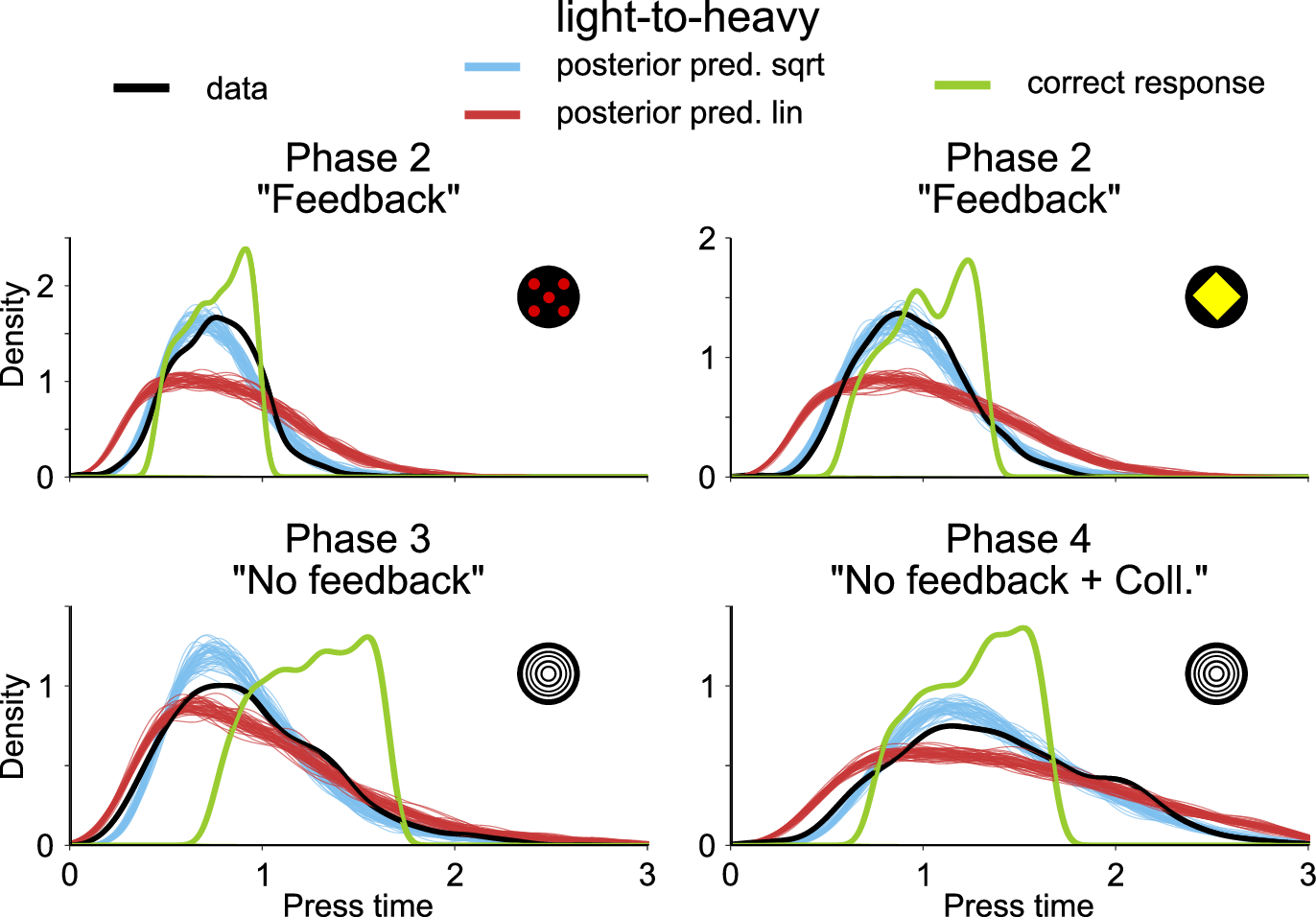

Supplement: S11 Fig — Posterior predictive for press times, actual press times and ideal responses for phases two to four and condition light-to-heavy. Black distributions show the actual data, red and blue ones display samples from posterior predictive distributions of both, the linear and Newtonian model, and green ones show the correct responses given perfect knowledge about the underlying physics and all parameters. Visualizing the enhanced suitability of this noisy Newtonian model framework compared to Newtonian models excluding prior preferences and uncertainties in describing human behavior. (TIF) [file pcbi.1007730.s012.tif]

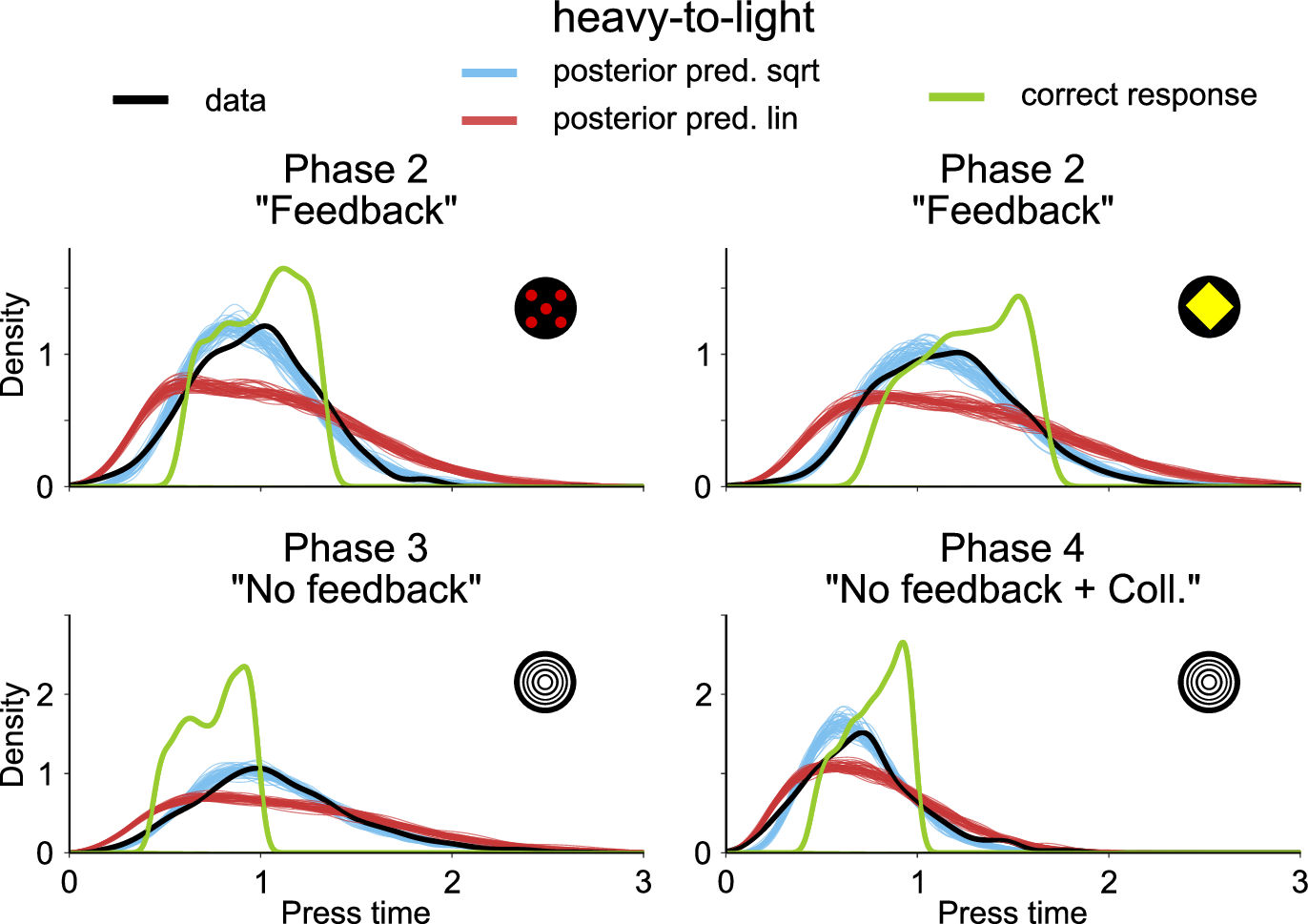

Supplement: S12 Fig — Posterior predictive for press times, actual press times and ideal responses for phases two to four and condition heavy-to-light. Black distributions show the actual data, red and blue ones display samples from posterior predictive distributions of both, the linear and Newtonian model, and green ones show the correct responses given perfect knowledge about the underlying physics and all parameters. Visualizing the enhanced suitability of this noisy Newtonian model framework compared to Newtonian models excluding prior preferences and uncertainties in describing human behavior. (TIF) [file pcbi.1007730.s013.tif]

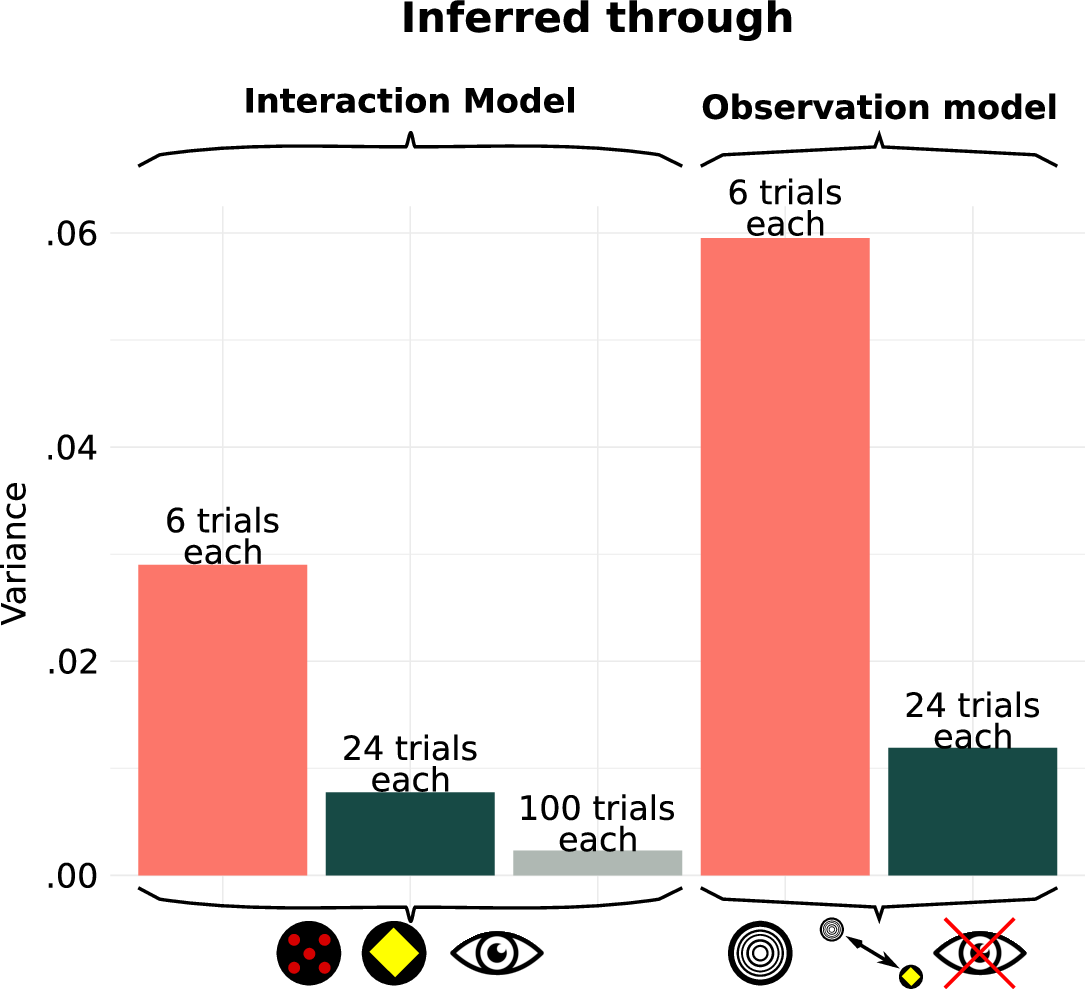

Supplement: S13 Fig — Barplot of averaged variance for both models and a given number of observations. First three columns show the average variance in posterior mass beliefs for inferences with 6, 24 and 100 trials per puck and participant. Two last columns show the average variance of mass beliefs of the unknown puck resulting from inference using the collision model for 6 and 24 trials, while using the posterior mass belief of the known pucks from the interaction model with 100 trials each. (TIF) [file pcbi.1007730.s014.tif]
