# Supplementary material for: Intuitive physical reasoning about objects’ masses transfers to a visuomotor decision task consistent with Newtonian physics
Source: PLoS Comput Biol. 2020 Oct 19;16(10):e1007730. doi: 10.1371/journal.pcbi.1007730 (PMC7647116; doi:10.1371/journal.pcbi.1007730)
Supplement: Attachment — Submitted filename: Response_Letter.pdf [file pcbi.1007730.s015.pdf]

Dear editors,
dear Ulrik and dear Wolfgang,

thank you very much for the generally very positive assessment of our manuscript. We have gone through all comments of the three reviewers and your comments and provide here detailed responses to all points. In this document comments from the first round of reviews are in regular font and our responses are in bold font. Two new version of the revised manuscript have been uploaded to the editorial system as requested, one with red highlights denoting where the text has been changed and a clean version.

We hope to have addressed all points that have been raised satisfactorily and are looking forward to hearing your response.

Best wishes,
Nils Neupaertl, Fabian Tatai, Constantin Rothkopf

In the following you can find our responses to your comments.

While all the reviewers praise the rigour and quality of the paper, reviewer two is sceptical of the innovativeness.

Reviewer two was indeed skeptical about the innovativeness of the current study and did provide a number of references and arguments for this view. We have to admit, that we were surprised by this statement, as we see a difference between picking a MAP in an inference task modeled by an ideal observer compared to a generative model of actions involving a cost function and action variability. We have answered these concerns and hope to have clarified, why we think that our study goes beyond previous research. This essentially boils down to the role and modeling of actions.

We repeat here our answer to reviewer 2 regarding this point here:

“Thank you for asking this question. This makes it clear, that we did not explain the additional value of the current study well enough. You mentioned:
1) “showing that people can infer mass after observing physical events like collisions”,
2) “that they can make physical predictions based on knowledge of mass”,
3) “can take actions which help them gather information necessary to make these inferences”.
The present experiments investigate, whether people can infer masses from observing physical events, and whether they can carry out a continuous control task, in which they have to use the inferred masses and carry those over to a non-linear function. We fully agree that several aspects in our experiments confirm results that are, among others, shown in *Sanborn et al. 2013, Ullman et al. 2018 and Bramley et al. 2018.* However, in our view, there is a difference between the “action” of deciding between larger/smaller, lighter/heavier in a 2 AFC paradigm and a continuous control task. The literature in psychology distinguishes between judgement, decision making, and control. Computationally, these are also very different “actions”.

In Sanborn et al. 2013 subjects were given extensive verbal instructions including descriptions of densities and masses of objects and the actions consisted of forced choices, i.e. decisions.

In Ullman et al. 2018 subjects were given extensive instructions including interpretations of colors relating to masses and properties of objects being constant across individual worlds. Actions consisted of forced choice answers such as light/medium/heavy.

In Battaglia, Hamrick, & Tenenbaum, 2013 subjects again had to provide forced choice answers such as whether a stack of bricks would fall or in which direction.

In Hamrick et al. 2016 subjects also had to judge whether 3D generated towers will fall or not and which of two colors corresponds to heavier ones, which are both 2AFC tasks, as well.

The Bramley et al. 2018 is very interesting and relevant: thank you for pointing this study out! We reference this study in our manuscript now. The experiments are looking at subjects' interactions with simulated virtual scenes in order for subjects to learn about physical properties of objects. Here actions are quantified in how much the entropy in posteriors of physical properties such as mass are decreased by interventions compared to the scene unfolding without interventions. This is a measure of how much an intervention action reduced overall entropy over physical parameters. Because subjects were asked questions such as whether object A was heavier, lighter, or of the same mass as object B, all possible parameter settings corresponding to these three cases were then evaluated with respect to how much the entropy reduction affected the comparisons between these three cases. This description of subject's inferences is therefore based on an ideal observer, i.e. inference. No variability of actions was included, no cost functions for action selection. The actions were not modeled as stemming from any process or generative model but instead were compared on the basis of their consequences on an aggregated entropy measure for inferences. Thus, essentially, actions are not modelled at all. Instead, what is modeled is to what degree the consequences of actions are contributing to an average measure of uncertainty respective a forced choice task.

Distinct from Bramley et al. (2018) and Sanborn (2013)/Ullman (2018) we showed people's ability to not merely infer physical properties from events like collisions but to subsequently act in non-linear tasks consistent with the actual Newtonian laws of physics. Here, people had to comprehend two different physical interactions by utilizing their belief about the underlying generative model and further apply them to a control task.

Another key distinction is the continuous nature of people's decisions compared to the discrete set of answers (e.g. heavier, lighter, same) in both, the Bramley and the Ullman paper. So this type of experiment places different demands on the performing participants due to: a) the need to understand two different, not-explained, physical phenomena and to make correct inferences using prior concepts of two distinct generative models b) the mandatory presence of concepts explaining how information can be transferred quantitatively correctly between scenes, e.g. how an inference of a latent variable from certain physical observations can influence prior

beliefs in another distinct action-control task and c) the continuous nature of participants' responses.

We hope that this clarifies what we perceive as novel contributions and that the reviewer can appreciate these contributions. We have made several changes to the text explaining this view with the most explicit change in the discussion by including the following paragraph:

“Different from tasks requiring a forced choice response [1–4, 9, 11–13], participants in the current experiments provided a continuous action by pressing a button for variable durations. Therefore, it is not sufficient to model our participants' actions as in an inference task, e.g. by assuming that subjects choose a press-time on the basis of the mass belief with highest probability, i.e. that MAP. Instead, modeling continuous actions requires a cost function, which additionally incorporates people's variability in press-times. This is evident when comparing the press-times according to the different models considered here, see S1 Appendix, ”Deviations from fully-observed Newtonian physics and model predictions”. Remarkably, posterior means of masses best explaining our participants' press-times were closer to the true masses used in the pucks' simulations for the quadratic cost function compared to the other cost functions. Thus, the current study establishes that people's deviations from the predictions of Newtonian physics are not only attributable to prior beliefs and perceptual uncertainties but also to implicit cost functions, which quantify internal costs for errors due to participants' action variability.”“

The second question of reviewer 2 relating to the significance of cost function also concerns the fundamental role of actions. Our answer there clarifies, that fundamentally, perception and action are probabilistically intertwined. The reason is, that if one selects a single estimate from a posterior distribution, say the MAP, then proceeding to use the MAP to carry out an action, then this is in general provably suboptimal. Thus, doing perception and then action on top of an estimate, is provably suboptimal. As such, we again think, that going from a forced choice task to a continuous control task for which we provide a generative model of actions including a cost unction and action variability has scientific value. Particularly, as taking actions into account in this way shows that participants' mass beliefs were more accurate than not taking these into account.

We hope that this clarifies our view that the current manuscript with its analyses and its inclusion of a generative model of actions and a comparison of cost functions is indeed innovative. We also hope that this can be regarded as an adequate response to this point.

One suggestion by the reviewer is to ask for a Bayesian model that takes into account the subject inference process of the mass, as opposed to just model the inference of the distance and the action itself.

The hierarchical Bayesian model describing the selection of press-times selects a particular action by observing an initial distance of a puck, which involves perceptual uncertainty, then uses an internal model involving the uncertain belief of the mass of a puck, and then generates a press-time action by taking into account the known probabilistic variability of action generation and a cost function. Thus, the model does include uncertainty over the unobserved mass. By using the generative model of

press-times and conditioning it on subjects' data, we obtain the posterior beliefs over masses of our subjects from subjects' data.

To address this point, we now renamed the subsection "Model result" to "Interaction model result" and include an additional subsection entitled "Observation model results". We use both these models to quantify, how the mass beliefs of our participants changed over trials by comparing posterior mass beliefs after 6 and 24 trials, see Fig.9 and Fig. 22 in the supplementary material. This quantifies how well and how fast subjects could infer the latent masses from interacting with the two pucks in phase 2 of the experiment, when visual feedback was available. This also answers several further questions reviewer 2 had regarding the differences between performance in phases two and four.

The reason for addressing these points in this way is, that the design of the experiment did not explicitly measure subjects' beliefs after each trial, especially in phase four when 24 collisions were shown in a single block without testing subjects' beliefs at intermediate stages. Nevertheless, this allows a direct comparison with the progress of learning mass beliefs from collisions, see also our answer to the following question.

We hope that this is an adequate response, as this quantifies learning of mass beliefs by our subjects across trials in phase two and compares the learning to phase four.

Currently, each subject infers the mass of the virtual pucks independently from the model. Incorporating the inference of the mass within the current model may be difficult, but adding a separate section with a separate model for the inference of the mass seems less onerous, and would still add novelty.

Again, the hierarchical Bayesian model describing the selection of press-times selects a particular action by observing an initial distance of a puck, which involves perceptual uncertainty, then uses an internal model involving the uncertain belief of the mass of a puck, and then generates a press-time action by taking into account the known probabilistic variability of action generation and a cost function. Thus, the model does include uncertainty over the unobserved mass. By using the generative model of press-times and conditioning it on subjects' data, we obtain the posterior beliefs over masses of our subjects from subjects' data.

We have included a new model that infers mass beliefs from the observation of pucks' collisions, see section "Observation model results". This model follows previous models in the literature that were only modeling the inference process of relative mass ratios based on the observation of collisions. The inferred mass beliefs are compared to the resulting mass beliefs from interacting with the pucks, described in the section "Interaction model result". Thus, the two models together quantify how the belief of masses changed after 6 and 24 trials when interacting with pucks or when observing puck collisions. This provides an account of the larger uncertainty of beliefs after only observing puck's collisions compared to interacting with them. We explain the new model, analyzed subjects' data with it, report the inferred mass beliefs of subjects and discuss the differences between phase 2 and phase 4.

We hope that this additional model and the analyses we provide using this model, especially the comparison of mass beliefs after 6 and 24 trials in phase two with those of phase four address these issues satisfactorily.

In the following we respond to the comments of all three reviewers.

Reviewer #1: In this manuscript, the authors test whether physical knowledge transfers from visual inference to a motor planning task. They asked participants to hold down a button to launch a puck at a target, where the length of time the button was held was proportional to the velocity divided by the object mass. Participants progressed through four phases, from no feedback, to feedback with two objects of different masses, to no feedback with a new object, followed by showing that new object colliding with the old objects, then a final phase with no feedback. Crucially, participants adjusted their actions between the third and fourth phases to relatively accurately calibrate their push times for the new mass, even though they were only able to indirectly infer the mass through the collision observations. Thus, people appear to be able to transfer mass knowledge between visual and motor domains using relatively calibrated physics.

This paper demonstrates that people transfer physical knowledge across domains, a fact that has not been directly tested but is a prediction of recent theories that people have an Intuitive Physics Engine for generalizable physical reasoning. The experiment is relatively straightforward yet elegantly tests the main point of the paper, and the authors provide a thorough set of analyses and models to test their theories (to the point where multiple times I would think of supplementary analyses, then find them elsewhere in the manuscript). The only major comment I have is that while the authors do a good job showing mass transfer in this experiment, a secondary conclusion – that participants' responses were "Newtonian" – is less well supported by the data. However, overall I believe this is a strong paper.

We thank the reviewer for this overall positive assessment!

As for the strength of the conclusion about the behavior following Newtonian Physics, we discuss this point in the next paragraph.

The secondary conclusion of this paper – that participants behaved in a "Newtonian" way – appears to be primarily motivated by model comparisons that test a model that includes a square-root relationship between press time and distance (Newtonian) against a model that assumes this relationship is linear. However, there is only clear evidence that the relationship is a square-root in the experimental phase with feedback – after that phase there is weak evidence in favor of the Newtonian model, whereas in the first phase there is equivalently weak evidence for the *linear* model (Fig. 7). The authors address this in the discussion as possibly stemming from unclear relationships between button pressing and force (pg. 16-17) which then gets corrected rapidly once feedback is available. I find myself agreeing with this interpretation and see it as the most parsimonious explanation. But a less charitable reader might suggest that the relationship between press time and distance is not based on physics at all, but rather is a functional mapping learned rapidly in phase 2; the physical reasoning about mass might then be the only physical part that then gets bolted onto the functional mapping in a sort-of Newtonian way.

I can think of three ways of adapting the text that might ground these claims better: (1) not referring to the square-root model as "Newtonian" and making

clear that the Newtonian nature of physics is in the mass inference and transfer; (2) hedging with the Newtonian claims a bit more (e.g., the transfer is "consistent with Newtonian physics" as suggested by the title, but it's not necessarily true that "participants transferred… in accordance with Newtonian physics" as suggested by the abstract); or (3) putting a section in the discussion with the least charitable, most non-Newtonian explanation that the authors can think of as an alternate model not tested here.

We agree that in principle alternative explanations could be found for which we did not provide model comparisons. We also agree, that our conclusion that subjects behavior was consistent with Newtonian physics is not only based on the square root relationship but also on the fact that press-times scale linearly with mass but with the square-root of initial distance, that contextual cues of color must have been interpreted as differences in mass by our subjects, the speed of adopting the correct functional scaling after only a few trials of visual feedback, the inference of relative masses being based on observations of collisions without the influence of friction.

In our view, a very strong argument for an internal physical model comes from the fact, that in phase two of the experiment, in which subjects obtained visual feedback of their actions, the two pucks were only distinguished by a color cue. No indication of mass was given in this phase of the experiment. Thus, a single non-linear function was never available, but instead two non-linear functions for the two pucks had to be learned. These two functions could only be factored into a single non-linear function, if subjects interpreted red and yellow colors as meaning mass, implicitly.

The first and only indication of masses was given in the 24 sequences of collisions between pucks at the beginning of phase 4 of the experiment. Note also, that no collision between the pucks of phase two were shown. The data therefore mean, that subjects readily interpreted the color cues of the two pucks as meaning two different masses. Suppose subjects had learned press-times as two non-linear functions of distance for the two color cues within four to six trials for the two pucks in phase 2. This alone would be a remarkable feat. Then subjects would have observed the 24 collisions and would have had to infer the mass ratios, infer that color meant mass in phase 2, and factorize the two non-linear mappings from phase 2 to recover the common factor of the non-linear scaling of initial distances and apply that to the new puck.

To make this clearer, we added the following passage to the discussion:
"Thus, the observation of the pucks' dynamics over six trials was sufficient to adjust the press-times with the square-root of initial distance, but differently for the two pucks, see Fig. 2. Note that in phase two, subjects only had a contextual color cue distinguishing the two pucks. Therefore, subjects needed to learn two different functions relating the pucks' initial distances to the required press-times, one for each puck. This is a strong indication, that subjects readily interpreted the differences between red and yellow puck as being caused by the latent masses, which were by itself not observable."

We also added the following sentence to the 'Interaction model results' section:
"Note that this implies that subjects must have interpreted the dynamics of the red and yellow pucks in the second phase as stemming from objects' masses, as

otherwise a physically consistent transfer to a new puck would be very difficult to explain."

In principle, it is much more fundamentally difficult to exclude the possibility that "a functional mapping" was "learned rapidly". After all, Newtonian physics consists of functional relationships that overwhelmingly are rather simple including linear and quadratic or square-root relationships. How rapid is rapid? Would an inductive bias for learning certain functional relationships faster than others already qualify as an internal model of physics? Are internal models probabilistic generative models or some form of function approximation space? Nevertheless, the results mean, that subjects needed to learn two non-linear functions and interpret yellow and red as a continuous number that needed to be factored out to obtain a single function. This is a very strong indication of an internal model involving mass.

But, we do not make explicit claims at the implementational or algorithmic level of description. We were very careful not to make very strong claims about putative internal representations, cognitive architectures, or specific internal reasoning systems, e.g. we write in the conclusion explicitly: "While our results do not provide direct evidence for the notion of internal simulations of a physics engine [39], they also do not contradict them".

We have decided to follow your suggestion (2) and have altered the following passages:

Abstract: "… to a subsequent visuomotor control task consistent with Newtonian physics …"

Introduction: "… their actions consistent with the inferred relative masses …"

Model results: "… adjust their press-times consistent with Newtonian physics …"

Discussion: "Thus, subjects scaled their press-times consistent with Newtonian physics …"

"… puck shooting task consistent with Newtonian physics."

Alternately, it might be possible to show more evidence for mass transfer using Newtonian physics using an extension to the "inferred mass" analysis displayed in Fig. 8. It's particularly compelling that individuals' inferred mass is on average mostly calibrated to the actual mass. But it's not clear that this requires extrapolation with Newtonian mechanics – would it be possible to extrapolate the constant factor in the linear model to what it "should" be if the mass ratios were extracted appropriately from the pre-phase 4 observations, and compare this to the linear model fits for individuals? If participants' behavior deviated strongly from this value, then it suggests that people are extrapolating their action choices using Newtonian mechanics and not a linear heuristic.

Again, subjects had no indication that the color cues meant mass in phase two of the experiment. Additionally, if a nonlinear function of distance had been learned in

phase two, the scaling of mass is impossible to infer, because no quantitative scaling can be inferred by red versus yellow. Note also, that subjects did not see collisions between the two pucks from phase two so that the inference of relative masses between the red and yellow pucks needs to be attributed to an internal physical model.

Before phase four of the experiment, nothing in the experiment had been explicitly related to the physical quantity mass. This is in our view an important factor in the current experiments. Mass came only into play in the 24 collisions at the beginning of phase 4 of the experiment. Thus, even if subjects "only" learnt some sort of non-linear mapping with 4 to 6 trials, this means that not only such a mapping needed to be learned but additionally that the color cue had to be interpreted as mass, a factorization in the forces, masses and coefficients of friction had to be carried out and separated from the non-linear scaling of distance.

To make this clearer, we rephrase the following sentence in the introduction:
"A succession of four phases investigated, what prior assumptions subjects had about the relationships between their actions and physical quantities, whether they could learn to adjust their actions to different objects when visual feedback about their actions was available, whether they would interpret the differences in objects' behavior in accordance with physical laws, and whether they could transfer mass ratios inferred from observing collisions to adjust their actions accordingly."

Similarly, when explaining the fourth phase of the experiment we added the following sentence:
"As the two pucks from phase two of the experiment were only distinguished by color, such a transfer indicates that subjects had attributed the different dynamics to their masses in accordance with Newtonian physics."

Furthermore, we added the following passage to the discussion:
"Thus, the observation of the pucks' dynamics over six trials was sufficient to adjust the press-times with the square-root of initial distance, but differently for the two pucks, see Fig. 2. Note that in phase two, subjects only had a contextual color cue distinguishing the two pucks. Therefore, subjects needed to learn two different functions relating the pucks' initial distances to the required press-times, one for each puck. This is a strong indication, that subjects readily interpreted the differences between red and yellow puck as being caused by the latent masses, which were by itself not observable."

Finally, we now also include the posterior predictive press times in the supplementary figures 20 and 21 for the linear model for phases 2, 3, and 4 of the experiment. These plots show, that the if subjects had used the linear model with the parameters best describing the linear factor in the model, press-times would strongly deviate from the observed ones.

More minor comments include:
• In the author summary, beginning of the introduction, and final paragraph of the discussion, the authors make the point that systematic failures of physical reasoning might be "the consequence of perceptual uncertainties and partial knowledge of object properties." This is not quite true – many of the classical

errors in reasoning cannot easily be explained by fully accurate physics (in fact, this failure to model a classic error was what prompted Smith, Battaglia, Vul, 2018). Instead, the findings tend to suggest that these errors might rely on distinct systems of reasoning to the more calibrated physics engine used for perceptual-motor tasks. It's in these types of perceptual-motor tasks that errors appear to arise from uncertainty and partial knowledge.

We agree with the reviewer that some previous research has suggested that some tasks are in line with well calibrated physical reasoning while other tasks cannot be reconciled easily with this view. We are also aware of the Smith, Battaglia, Vul, 2018 study and we agree that this study shows clear differences in the accuracy of predictions between tasks. And, we also agree with the statement: "there are also differences between these experiments in how this knowledge is measured: studies that demonstrate failures of physical knowledge tend to rely on asking participants to draw the future trajectory of objects or verbally explain how the world will unfold, while studies that show accurate knowledge tend to require people to make single judgments about a continuous physical property such as weight or future location."

However, in our view it is not clear, what the root cause of these differences is, i.e. how a computational model could describe the reasons for these differences across tasks quantitatively. Do human participants reason differently given different tasks? Does this mean that different models are used, or different parameters, or different representations, or different priors, or different actions, or different cost functions? Or are these differences not captured by any of these modeling components and something else needs to be brought to the modeling? Thus, that humans "employ different modes of physical reasoning depending on the format of the task" may be one possible explanation, but it is not clear, how this can be formalized. Similarly, it may be possible, that humans "rely on different sources of knowledge" but again, it is not clear, how to formalize this.

We have made the following changes in the text to account for this discussion:

Author summary:
"While people are known to show multiple systematic errors when reasoning about Newtonian physics, recent research has provided evidence that some of these failures can be attributed to perceptual uncertainties and partial knowledge about object properties."

Introduction:
"Nevertheless, some persistent failures of intuitive physical reasoning have been suggested to be caused by distinct systems of reasoning compared to the more calibrated physical reasoning underlying visuomotor tasks [15]."

Discussion:
"It is only more recently, that a number of these deviations have been explained through computational models [9–12,39] involving structured generative models relating observed and latent variables probabilistically."

• The authors present the number of trials used in Phase 1 and Phase 2 (pg. 6), but I was unable to find the corresponding information for Phases 3 and 4

Yes, thank you for catching this. Subjects performed one hundred trials each (phase 3 & 4). We added this in the 'Experimental design and data':
"Subjects were asked to shoot a new puck they had not seen before to 199 the target without visual feedback, as in the first experimental phase, for one-hundred 200 trials (Fig. 1 B, No Feedback)."

• The error scale in Fig. 2B might mislead readers by making more erroneous points lighter and therefore less salient. Since this information is already present in the graph (as distance from the blue line), it might be better to use a constant alpha here.

We have removed progressive transparency of data points as a function of distance to the blue line and hope to have improved the interpretability of the graph.

• On pg. 8, the authors report correlations between distance and press times or square-root press times – what are readers to make of the fact that they are nearly identical in all four phases? It strikes me that many of the stimuli sit in a range where the quadratic function looks nearly linear (Fig. 3), and therefore my take-away is that people are modulating their press times with distance, but it's not particularly diagnostic here of the exact functional relationship. This might also affect the statement on pg. 9, "analyses provided initial evidence, that subjects scaled their press-times… with a square-root function of initial distance"

Yes, we fully agree with the reviewer in terms of the conclusions that these correlations allow. Nevertheless, the correlations provide a first, albeit very weak indication, that subjects scaled their press-times as a linear function of initial distance in the first phase of the experiment, when no visual feedback had been obtained yet. By contrast, after having obtained visual feedback, the square-root relationship had a slightly higher correlation value in phases 2 to 4. The main point for including them in our view, is that they provide an important motivation for analyzing the data with a probabilistic generative model instead of a correlation analysis.

"Taken together, these analyses suggest, that subjects adjusted their press-times both depending on the distance of the pucks to the target and depending on the pucks' masses used in the simulation. Furthermore, the analyses provide a very weak initial hint, that subjects may have scaled their press-times with respect to mass and with a non-linear function of initial distance after having obtained visual feedback about the pucks' motion. Finally, observing collisions between pucks lead subjects to adjust their press-times even without obtaining visual feedback. In the following section we provide two computational generative models, one for the sliding task and one for the collision observation task to quantitatively analyze participants' press-times in terms of perceptual, physical, and behavioral quantities.

• In Fig. 4 (pg. 10), values for model parameters are displayed, some of which are later fit and found to have little individual variability (sigma_x), or fit at an individual level (sigma_j^t). However I could not tell how the m_j,k parameter is fit, and sigma_j^t values don't match up with those shown in Fig. 6. How were

these values chosen? Are they meant to map on to the actual values used in the model?

Thank you for the question. Fig. 4 shows the values of the parameters determining the prior distributions for each latent variable. Fig. 6 instead shows the resulting posterior distributions after inference, i.e. results from running the model given the actual behavioral data and the described prior distributions. Similarly, individual values reported later in the text refer to posterior modes.

The only fixed parameter is sigma_x. We initially had also used a prior probability distribution for this latent parameter but our inference consistently showed a small value of 0.05m, which showed very little variability across subjects. Therefore, we decided to fix this value.

To make this clearer, we added the following text to the caption of figure 4:

"Fig. 4 Hierarchical Bayesian network for the Newtonian model class. The model expresses the generative process of observed press-times t^{pre} across trials i, participants j, and i,j pucks k including Weber-Fechner scaling given perceptual uncertainties of distance xi,j and mass mj,k of the pucks and subjects' press-time variability. The parameter values refer to the prior probability distributions. See the text for details."

- **On pg. 11 for Eqs. 3 & 4, it could be useful to reiterate that these relationships only hold for a constant puck mass**

Yes, thank you, we now explicitly mention in the text: "for each object which is associated with a constant mass".

- **The sentence starting on pg. 12, line 396 might be more grammatical to start "The data show highly significant…"**

Yes, thank you.

- **The sentence on pg. 13, line 412 should start "[An] ANOVA…"**

Yes, thank you.

- **The sentence on pg. 14, line 452 should either be connected with the last sentence with a comma, or start "This results in lower…"**

Yes, thank you. We used the former suggestion.

- **In Fig. 11, which line represents the feedback vs. no feedback conditions?**

Thank you for pointing this out. We have added a legend clarifying which is which: “Red lines represent CDF of phase 2 (feedback) and black CDF of phase 3 (no feedback).”

• The data does not appear to be available on the authors’ lab website, but should be posted before publication

Yes, absolutely, we will make the data available if the manuscript gets accepted.

Reviewer #2: This paper examines the link between physical reasoning and visuomotor control. They perform an experiment in which participants press a button to launch pucks of varying mass at a target location with and without visual feedback. The experimental results reveal that participants quickly learn to appropriately control the puck, and that they can infer the mass of a new puck by just watching videos of it colliding with other pucks, and that they can use this knowledge of mass to more accurately control it. Through a Bayesian analysis, it is demonstrated that participants accurately estimate the mass and that they control the pucks according to Newtonian laws (i.e., by pressing the button for a duration that is a function of the square root of the distance it needs to travel) while also optimizing for internal costs/preferences.

Overall, I found this an enjoyable paper to read and believe it to be a useful contribution to the literature on intuitive physical reasoning, especially in terms of confirming a link between inference of physical properties and control. I found the paper relevant in that it supports the hypothesis that people ought to use their knowledge of physical properties to guide behavior. Moreover, it is extremely rigorous and thorough in demonstrating its results.

We thank the reviewer for this overall favorable evaluation of our manuscript.

However, I thought there were three main shortcomings:

1. I do not feel that the paper contributes substantially new knowledge beyond that which already exists in the literature. There have been a number of other papers at this point showing that people can infer mass after observing physical events like collisions (e.g. Sanborn et al. 2013; Ullman et al. 2018), that they can make physical predictions based on knowledge of mass (Battaglia, Hamrick, & Tenenbaum, 2013; Hamrick et al. 2016), and that they can take actions which help them gather information necessary to make these inferences (Bramley et al. 2018) in accordance with Newtonian laws. It is perhaps then not too surprising at this point that people take simple control actions based on their knowledge of mass too. Having confirmed this is useful, but in light of the prior body of work I am not sure how significant this insight is.

Thank you for asking this question. This makes it clear, that we did not explain the additional value of the current study well enough. You mentioned:
1) “showing that people can infer mass after observing physical events like collisions”,
2) “that they can make physical predictions based on knowledge of mass”,
3) “can take actions which help them gather information necessary to make these inferences”.
The present experiments investigate, whether people can infer masses from observing physical events, and whether they can carry out a continuous control task, in which they have to use the inferred masses and carry those over to a non-linear function. We fully agree that several aspects in our experiments confirm results that are, among others, shown in *Sanborn et al. 2013, Ullman et al. 2018 and Bramley et al. 2018.* However, in our view, there is a difference between the “action” of deciding between larger/smaller, lighter/heavier in a 2 AFC paradigm and a continuous control

task. The literature in psychology distinguishes between judgement, decision making, and control. Computationally, these are also very different “actions”.

In Sanborn et al. 2013 subjects were given extensive verbal instructions including descriptions of densities and masses of objects and the actions consisted of forced choices, i.e. decisions.

In Ullman et al. 2018 subjects were given extensive instructions including interpretations of colors relating to masses and properties of objects being constant across individual worlds. Actions consisted of forced choice answers such as light/medium/heavy.

In Battaglia, Hamrick, & Tenenbaum, 2013 subjects again had to provide forced choice answers such as whether a stack of bricks would fall or in which direction.

In Hamrick et al. 2016 subjects also had to judge whether 3D generated towers will fall or not and which of two colors corresponds to heavier ones, which are both 2AFC tasks, as well.

The Bramley et al. 2018 is very interesting and relevant: thank you for pointing this study out! We reference this study in our manuscript now. The experiments are looking at subjects’ interactions with simulated virtual scenes in order for subjects to learn about physical properties of objects. Here actions are quantified in how much the entropy in posteriors of physical properties such as mass are decreased by interventions compared to the scene unfolding without interventions. This is a measure of how much an intervention action reduced overall entropy over physical parameters. Because subjects were asked questions such as whether object A was heavier, lighter, or of the same mass as object B, all possible parameter settings corresponding to these three cases were then evaluated with respect to how much the entropy reduction affected the comparisons between these three cases. This description of subject’s inferences is therefore based on an ideal observer, i.e. inference. No variability of actions was included, no cost functions for action selection. Importantly, the actions were not modeled as stemming from any process or generative model but instead were compared on the basis of their consequences on an aggregated entropy measure for inferences. Thus, essentially, actions are not modelled at all. Instead, what is modeled is to what degree the consequences of actions are contributing to an average measure of uncertainty respective a forced choice task.

Distinct from Bramley et al. (2018) and Sanborn (2013)/Ullman (2018) we showed people’s ability to not merely infer physical properties from events like collisions but to subsequently act in non-linear tasks consistent with the actual Newtonian laws of physics. Here, people had to comprehend two different physical interactions by utilizing their belief about the underlying generative model and further apply them to a control task.

Another key distinction is the continuous nature of people’s decisions compared to the discrete set of answers (e.g. heavier, lighter, same) in both, the Bramley and the Ullman paper. So this type of experiment places different demands on the performing participants due to: a) the need to understand two different, not-explained, physical phenomena and to make correct inferences using prior concepts

of two distinct generative models b) the mandatory presence of concepts explaining how information can be transferred quantitatively correctly between scenes, e.g. how an inference of a latent variable from certain physical observations can influence prior beliefs in another distinct action-control task and c) the continuous nature of participants' responses.

We hope that this clarifies what we perceive as novel contributions and that the reviewer can appreciate these contributions. Additionally, this also relates to your second point, which we answer in the following. We have made several changes to the text explaining this view with the most explicit change in the discussion by including the following paragraph:

"Different from tasks requiring a forced choice response [1–4, 9, 11–13], participants in the current experiments provided a continuous action by pressing a button for variable durations. Therefore, it is not sufficient to model our participants' actions as in an inference task, e.g. by assuming that subjects choose a press-time on the basis of the mass belief with highest probability, i.e. the MAP. Instead, modeling continuous actions requires a cost function, which additionally incorporates people's variability in press-times. This is evident when comparing the press-times according to the different models considered here, see S1 Appendix, "Deviations from fully-observed Newtonian physics and model predictions". Remarkably, posterior means of masses best explaining our participants' press-times were closer to the true masses used in the pucks' simulations for the quadratic cost function compared to the other cost functions. Thus, the current study establishes that people's deviations from the predictions of Newtonian physics are not only attributable to prior beliefs and perceptual uncertainties but also to implicit cost functions, which quantify internal costs for errors due to participants' action variability. "

2. While the modeling component of the paper is as a whole impressively precise and rigorous, it does not innovate much beyond past work either, except for the introduction of (1) motor variability and (2) a cost function. The motor variability component is a straightforward addition to existing Bayesian models. The cost function seems interesting, but I was confused about where the form of the cost functions come from. I looked at how they are defined in the appendix, but it still is not clear to me. For example, isn't the 0-1 cost supposed to be discontinuous? Why then is it represented by a lognormal distribution? Why are there differences in choosing t^int as MAP/mean/median? Perhaps these are standard in the literature on visuomotor control, but this is not my area of expertise and so it would be helpful to readers like myself to derive where these cost functions come from. Moreover, while it is interesting that the quadratic cost function seems to work best, I do not have a good intuition for why this is or what it means, and the paper does not really attempt to provide an intuition. It would be helpful to provide more discussion on what the quadratic cost function implies and what its implications are.

Yes, we can gladly go into more detail about the integration of costs in the modelling of participants' responses. The cost function in general is not restricted to visuomotor control but stems from Bayesian decision theory. Say you have a posterior over some latent variable, e.g. describing a property of the world you want to infer. What value should you pick as your decision? As an example, what should your percept of

a latent property of a visual scene be, given a posterior pdf? A pdf does not tell you how to decide. In Bayesian decision theory, a cost function tells you in a principled, normative way how to decide, given a posterior.

Often, the cost function is omitted, especially when perceptual uncertainties are conveniently modeled as Gaussians. For the Gaussian distribution, the mean, mode, and median coincide. But we chose a lognormal distribution to reflect the variability in responses for three reasons: 1) we can explain and model uncertainties for each participant, 2) we can account for Weber-Fechner like phenomena, that are commonly found in psychophysical tasks and continuous action control tasks and 3) samples arising from this distribution are strictly positive, which is fundamentally important when dealing with press times. Thus, the lognormal is better suited for our model requirements than the simple and widespread Gaussian distribution.

Another feature of the lognormal distribution, which differs from the Gaussian, is its asymmetry and the reason, why different cost functions can be distinguished. Each of these cost function we used leads to different optimal solutions for responses: 0-1 costs are optimized by aiming for the mode of a distribution, absolute cost by the median and quadratic cost by the mean. These solutions do not differ for the symmetrical Gaussian distribution. However, they do differ for asymmetrical lognormal distributions. Thus, we can compare these cost functions using modeling approaches with differing solutions and evaluate which of these models explains the data best. It is absolutely crucial to integrate cost functions when explaining actions. Without any cost function models rather explain the perception than the action itself. However, when there is no explicit consideration of a cost function in a model, this does not necessarily mean that there is no cost implemented. As mentioned before, the choice of distributions and as well as their structure already defines whether differing cost functions can be distinguished. So, here, in order to evaluate people’s behavior more nuanced we at least incorporated three different potential candidates.

The current study provides also a further argument for including cost functions in these types of analyses. The different cost functions influence the inference of our subjects’ mass beliefs: the quadratic cost function, for which we found evidence, leads to the mass beliefs being much more accurate compared to using the other two cost functions. As we wrote:
“Thus, while previous research has demonstrated that human biases and systematic deviations from the predictions of Newtonian physics are attributable to perceptual uncertainties and prior beliefs, the present experiments and analyses provide evidence that costs functions implicit in subjects’ behavior also need to be taken into account for explaining such deviations.”

We have published a number of papers, in which we show how to infer cost functions and in which we show, that behavior is better accounted for when implicit cognitive costs are modeled explicitly. If you are interested, here some references:

C. A. Rothkopf, D. H. Ballard: 'Modular inverse reinforcement learning for visuomotor behavior', Biological Cybernetics, 107(4), 477-490, 2013

D. Hoppe, C. A. Rothkopf: 'Learning rational temporal eye movement strategies', Proceedings of the National Academy of Sciences (PNAS), 113(29), 8332-8337, 2016

F. Schmitt, H.-J. Bieg, M. Herman, C. A. Rothkopf: 'I See What You See: Inferring Sensor and Policy Models of Human Real-World Motor Behavior', Thirty-First AAAI Conference on Artificial Intelligence (AAAI-17), 2017

D. Hoppe, S. Helfmann, C. A. Rothkopf. Humans quickly learn to blink strategically in response to environmental task demands. Proceedings of the National Academy of Sciences (PNAS), 2018

R. Zhang, S. Zhang, M. H. Tong, Y. Cui, C. A. Rothkopf, D. H. Ballard, M. M. Hayhoe. Modeling sensory-motor decisions in natural behavior. PLoS Computational Biology, 14(10), 2018

D. Hoppe, C. A Rothkopf. Multi-step planning of eye movements in visual search. Scientific Reports, 9(1):144, 2019

Finally, including actions to an inference process is rather tricky. The reason is, that if one selects a single estimate from a posterior distribution, say the MAP, then proceeding to use the MAP to carry out an action, then this is in general provably suboptimal. Thus, doing perception and then action on top of an estimate, is provably suboptimal. This is the reason, why we used a generative model of the task involving perception and action. Note that in our model actions are selected not based on a point estimate but on the basis of the full model including the respective probability distributions and functional relationships between variables.

We have revised the paragraph introducing the cost function as follows and hope to have clarified its role:
“However, modeling perception as inference may not be sufficient to describe our participants’ behavior and their selection of actions. Given a posterior over mass and distance describing the perceptual belief of a subject on a particular trial, a specific press-time needs to be selected. In order to model this selection process we take action variability and potential cost functions into account. Cost functions govern which action, here the press-time, should be chosen given a posterior belief and a cost function, which quantifies how the decision process penalizes errors on the task. This means that it is assumed that participants select an action that minimizes potential costs associated with missing the target. Loss functions, describing the rewards or costs for every action in the action space, can have any arbitrary form, nonetheless we chose a set of three standard loss functions and compare their predictions: 0-1, absolute and quadratic loss functions. These three canonical loss functions express subjects’ implicit preferences for reaching a decision about press-times based on a putative perceptual posterior: the 0-1 loss corresponds to penalizing equally all deviations between the chosen value and the correct value, the absolute loss corresponds to penalizing deviations from the true value linearly, and the quadratic loss penalizes the deviations quadratically. It can be shown that these loss functions lead to different decisions for a continuous variable with a non-symmetric distribution [31]. Thus, assuming that humans do have costs for missing the target and associated policies to minimize these costs, leads to three different model versions for each model class (see S1 Appendix, ”Implementation of cost functions”).”

3. I was somewhat disappointed to see that the Bayesian model did not try to capture how people integrate the evidence that they obtain to infer the relevant

physical properties. In particular, I felt that one of the most interesting results was that while it seems that people do get some evidence from watching the collisions, this evidence is not as strong as actually getting to see feedback after taking an action. It's not totally clear to me why this is, and I strongly suspect an ideal Bayesian learner would not capture this difference---especially since it seems within 6 trials of action-feedback people have gotten all the evidence they need, while after 24 trials of watching collisions they still haven't fully inferred the mass. I think the paper could be much more impactful if it could show not just that people make an inference about mass and use it for control, but how they make these inferences.

We agree with the reviewer that, in principle, modeling the learning process is indeed interesting. But a full learner model is beyond the scope of this manuscript. The reason is, that modeling learning is rather straightforward when only inferences are concerned, but much more computationally involved, when actions are involved. Nevertheless, we decided to address the discrepancy in beliefs after the interaction task in phase 2 and after observing the collisions in phase 4 by including a second hierarchical Bayesian model, which describes the inference based on the observations. This allows comparing the reduction of uncertainty after 6 and 24 trials both when interacting with pucks and when observing pucks. We include an analysis of the change in participants' beliefs using the two models.

It is correct that inferences made on basis of the observation of these 24 collisions does not mirror the precision of inferences through feedback. Especially, when comparing the fact, that participants were able to achieve an equilibrium of mean performance during phase two within 6 trials. However, in order to understand this difference, we have to look at the possible reduction in uncertainty that participants could achieve during the different phases of the experiment. In phase 2 participants received richer information, as precision depends on perceptual uncertainty, which was very low across all subjects, about the outcome of their action and could adjust their behavior after each trial. Over the course of 200 trials participants could refine their responses, but as we showed in the change-point analysis, they already reached an equilibrium after 6 trials. Differently, before propelling the pucks in phase 4 participants watched 24 collisions in one single block. Their inferences thereby relied on their previously inferred beliefs about the pucks they interacted with in the feedback phase, on their perception of incoming and resulting velocities of these pucks and, of course, on their generative model, which they are using to explain their observations. This clarifies that inferences passed on the observation of collisions suffer from the perceptual uncertainty about velocities before and after these elastic collisions.

Given your request, we decided to include a second modeling part in the manuscript. We renamed the section "Model result" to "Interaction model results" and added the section "Observation model result". The hierarchical Bayesian model is depicted in fig. 9, middle panel. Latent variables are shown as white, observed ones as grey nodes. Note that this model explains the inference process from the experimenter's point of view. Running this model with the known initial and resulting velocities, as well as with the inferred belief about the pucks from phase 2 on a subject-by-subject basis we can infer a posterior belief about the unknown puck's mass. Further we introduce uncertainty to the perception of velocities with a fix value of sigma at 0.2 for a log-normal distribution with its mode at the actual velocity (for comparison see fig. 6

in Sanborn, A. N., Mansinghka, V. K., & Griffiths, T. L. (2013). Reconciling intuitive physics and Newtonian mechanics for colliding objects. *Psychological review*, *120*(2), 411, or section “Subject Performance” in K. A., & Vul, E. (2013). Sources of uncertainty in intuitive physics. *Topics in cognitive science*, *5*(1), 185-199).

Using this model of the observation of collisions we can now infer subjects' unknown, latent belief about the new puck, as shown in the right panel of fig. 9. In addition, we can compare uncertainties of mass beliefs in both models. We ran the hierarchical interaction model on data from the feedback phase 2 in order to infer mass beliefs based on the first 6, 24 and all 100 trials per puck. Resulting averaged variances are shown in fig. 22 within the first three columns. Subsequently, we used the mass beliefs of phase 2 inferred with all 100 trials as input (grey) for the Collision model to infer beliefs for the new puck in phase 4. The assumption is, that participants' mass beliefs were uncertain even after 100 interaction trials. Note, that the bar plot shows the variances in beliefs. Given the structure of the observation model, the uncertainty of mass beliefs of the red and yellow pucks from the end of the phase 2 strongly influences the quality of potential inferences. More specifically, the uncertainty in these mass beliefs acts as an upper bound for inference precision in phase 4. As shown in the last two columns, the average uncertainty about the unknown puck's mass in phase 4 is larger than the uncertainty in the mass belief input from phase 2 used for its inference (Fig. 22, grey column, 100 trials).

Thus, the lower precision of participants' mass beliefs in phase 4 is due to lack of precise knowledge about the feedback pucks' mass and their uncertain sensory measurements with regards to their perception of puck velocities. We also note the caveats of this model:
“Furthermore, the predictions of the idealized observation model deviate quantitatively from mass beliefs inferred using the interaction model for two reasons: 1) participants would need to remember their belief about the mass of feedback pucks perfectly while performing in phase 3 and 4, whereas these beliefs may well suffer from memory effects, and 2) they will be biased from initial, uninformed guesses in phase 3 before they have seen any collisions and thus probably show recency effects, as well (see e.g. participant 7 & 8 in Fig.19).”

Thus, while I think this work merits publishing, I think its potential impact is currently limited. I think that if the paper showed how physical knowledge played into more complex control tasks, or if it could more comprehensively model the entire process of learning, inference, and control, then it would be much stronger. At a minimum, I think the paper needs to do a better job of explaining how the cost functions work.

Looking at the literature, the vast majority of physical reasoning papers use forced choice tasks under the Gaussian assumption and very few use interaction-tasks. Of those that use interaction-tasks, very few model the required actions quantitatively in terms of the perceptual uncertainties, underlying world-model, motor variability, and cost function, as an action is not identical to picking a MAP from a Gaussian posterior. In the current study, we provided a full generative model of how to select an action. Therefore, as laid out above, in our view, the current experiments and modeling constitute a scientific contribution. A computational model involving perceptual uncertainty, action uncertainty and learning corresponds to a POMDP and is among the most notoriously complex computational problems. This constitutes a

major challenge which we think can be deferred to future research. As such, we hope that the current manuscript does contribute to our current knowledge.

I also have a few miscellaneous comments:
- Sentences should not start with “e.g.” (lines 40, 45, 72, 76)

We have change the text as follows:
“It has been quite successful at explaining a range of discrepancies between predictions of Newtonian physics and human predictions for various perceptual inference tasks, including subjects’ biases in judgements of mass ratios when observing simulated collisions of objects, if perceptual uncertainties are taken into account [9,15]”

“As an example, this suggests an explanation for the fact that judgements about physical situations based on a static image representing a situation at a single timepoint have usually been reported to deviate more from physical ground truth compared to richly animated stimuli [17], which additionally allow to estimate objects’ velocities.”

“Specifically, when sliding an …”

“Specifically, while it is known that subjects …”

- I think Zago & Lacquaniti’s work warrants slightly more discussion than it is given (even if just to say that their work does not focus on inferring unobservable quantities like mass)

Thank you, we added the sentence to the introduction:
“Nevertheless, these studies did not investigate the inference and reasoning of unobservable physical quantities.”

- It is probably also worth citing Ullman et al. (2018) and Bramley et al. (2018). The first paper models how people infer unobservable physical quantities and the second paper explores how people select actions to discover information about unobservable physical quantities.

Yes, thank you for pointing these out. We have cited both papers throughout the text.

- I did not understand why friction was removed in the collisions in phase 4. Doesn’t this actually make the task harder, since participants would have to jointly infer that friction had changed and infer the mass of the new puck? It would be good if this choice could be further justified.

If collisions were simulated with friction, participants would have been able to observe the new puck gliding and interacting with the surface and its friction. This would have been an additional cue for subjects. However, we wanted to study, whether participants can utilize inferred mass ratios and transfer their inferences to a task, in

which masses have to be scaled linearly and the initial distances have to be scaled with a square root function due to friction. Thus, observing the collisions without the influence of friction ensures that subjects transferred the inferred mass and that they could not use cues from the dynamics during gliding, e.g. in some sort of simple function approximation scheme.

We have added a clarifying sentence in the description of phase 4:
"Note that the pucks were simulated without the influence of friction in these collisions, ensuring that participants only obtained a cue about relative masses and not about the dynamics under friction for the puck from phase 3."

- I don't understand what the numbers 1, 2, and 3 are referring to in Figure 2a.

Sorry for not being clear here. We used the visible rings of the target to discretize the performance of this continuous action control task. Number 1 marks the bullseye, 2 the most inner ring and so on, enabling us to visualize the performance in a simplified and thus lucid manner. We have added this to the figure's caption:
"The number of the ring at which the center of the puck stopped was used for coding performance, e.g. 1 and 3 in the shown cases."

- Lines 236-243: I was initially confused because it seemed to me based on correlation that you could not distinguish between linear vs. square root relationships here. Indeed, it is not until later in the paper that I realized that the way this is distinguished is with the Bayesian model. It would be helpful to mention here that these two hypotheses cannot yet be distinguished but that you will do that later in the modeling section.

We added a sentence at the end of the paragraph:
"In the following section we provide a computational generative model of the sliding task to quantitatively analyze participants' press-times in terms of perceptual, physical, and behavioral quantities."

- What was the mean absolute error in phase 1, and how does it compare to phase 3?

In phase one, subjects could not see the consequences of their button presses. Thus, there is no error and no absolute error in phase 1 because in this phase no mass was assigned to the puck. Participants were not able to learn anything about this proxy object, which was never simulated moving. The sole purpose of phase one was to obtain an indication of the assumptions that subjects brought to the experiment. Therefore, the most probable mass beliefs were computed on basis of the initial distance – see fig. 17 & 18.

- Lines 277-279: are these results reversed? "Approximately" is used to describe the results for the light-to-heavy condition, where the p value is listed as <0.001, and for the heavy-to-light condition the results are stated more strongly even though the p value is >0.05.

They are not reversed. The Kolmogorov-Smirnov test aims at differences between two one-dimensional probabilities and thus the non-significant p-value at .092 shows that the press-time distributions in phase two and three do not significantly differ for the ‘heavy-to-light’ condition. The test for the ‘light-to-heavy’ condition yields a significant difference, however with a small difference (D= .156), additionally see fig. 11 in the appendix.

- Line 299: I am not sure this claim is supported by the evidence presented so far. As far as I can tell, the analyses presented at this point in the paper do not distinguish between linear and square root relationships. Also, I could not find where it was tested that response times scaled linearly with mass at all.

Yes, we fully agreed also with reviewer 1 in terms of the conclusions that these correlations allow. The correlations provide a first, albeit very weak indication, that subjects scaled their press-times as a linear function of initial distance in the first phase of the experiment, when no visual feedback had been obtained yet. By contrast, after having obtained visual feedback, the square-root relationship had a slightly higher correlation value in phases 2 to 4. But the main reason for including them is that they provide a motivation for analyzing the data with a probabilistic generative model instead of a correlation.

We have changed the text in the following way:
“Furthermore, the analyses provide a very weak initial hint, that subjects may have scaled their press-times with respect to mass and with a non-linear function of initial distance after having obtained visual feedback about the pucks’ motion. Finally, observing collisions between pucks lead subjects to adjust their press-times even without obtaining visual feedback. In the following section we provide two computational generative models, one for the sliding task and one for the collision observation task to quantitatively analyze participants’ press-times in terms of perceptual, physical, and behavioral quantities.”

Regarding the linear scaling of response times: thank you for catching this! Indeed, we did not test for this, as the masses are never available to our subjects. Instead, masses are inferred quantities and therefore dependent on our subjects’ interpretation of different dynamics of pucks as mass or the inference in our generative model. Therefore, we changed the text:
“Furthermore, the analyses provide very weak initial evidence, that subjects scaled their press-times with respect to mass and with a non-linear function of initial distance after having obtained visual feedback about the pucks’ motion.”

- Equations 3 and 4: should these not have a term for mass in them?

Yes, of course, it is true, that mass is a crucial scaling factor for the final equations as the other variables in equation (1). Here, we focused on the functional relationship between the press-time and distance, and therefore we omitted the remaining variables, leading to the proportionality sign instead of an equality sign.

- Line 396: Are these corrected for multiple comparison? If so, please report the correction used. If not, please use a correction here.

Thank you for catching this! Now, we report the correlation and their p-values (if there is a significant correlation, anyway) as well as the three comparisons of the linear and the square-root model for each cost function with p-values each <.001. The critical alpha value of .017, Bonferroni-corrected for these three comparisons, is now reported as well. We reported here the p-values and the associated correction.

“The data show highly 429 significant correlations for each model ($p < 0.001$; 0-1 loss function: $\rho_{New} = 0.167$, 430 $\rho_{lin} = -0.550$; abs. loss function: $\rho_{New} = 0.124$, $\rho_{lin} = -0.643$; quadratic loss function: 431 $\rho_{New} = 0.0976$, $\rho_{lin} = -0.686$) and higher correlation in the linear model for each cost 432 function ($p < 0.001$, with Bonferroni corrected $\alpha_{crit} = .017$).”

- I thought Fig. 19 & 20 were quite interesting that that they should be highlighted more in the main text---they are alluded to in Lines 614-617 but I think they should be referenced more explicitly!

Yes, thank you. We also think that these are quite interesting, as they can provide additional evidence for the importance of distinguishing between perfect knowledge, function approximation, and the consequences of modeling actions with their variability. We have now included the predictions when using the linear model and reference these explicitly in the discussion in the paragraph highlighting the modeling of actions in this study.

“Different from tasks requiring a forced choice response [1–4, 9, 11–13], participants in the current experiments provided a continuous action by pressing a button for variable durations. Therefore, it is not sufficient to model our participants’ actions as in an inference task, e.g. by assuming that subjects choose a press-time on the basis of the mass belief with highest probability, i.e. that MAP. Instead, modeling continuous actions requires a cost function, which additionally incorporates people’s variability in press-times. This is evident when comparing the press-times according to the different models considered here, see S1 Appendix, ”Deviations from fully-observed Newtonian physics and model predictions”.”

References

Battaglia, P. W., Hamrick, J. B., & Tenenbaum, J. B. (2013). Simulation as an engine of physical scene understanding. Proceedings of the National Academy of Sciences, 110(45), 18327-18332.

Bramley, Gerstenberg, Tenenbaum, and Gureckis (2018). Intuitive experimentation in the physical world. Cognitive Psychology, (195), pp. 9–38

Hamrick, J. B., Battaglia, P. W., Griffiths, T. L., & Tenenbaum, J. B. (2016). Inferring mass in complex scenes by mental simulation. Cognition, 157, 61-76.

Sanborn, A. N., Mansinghka, V. K., & Griffiths, T. L. (2013). Reconciling intuitive

physics and Newtonian mechanics for colliding objects. Psychological review, 120(2), 411.

Ullman, Stuhlmüller, Goodman, and Tenenbaum (2018). Learning physical parameters from dynamic scenes. Cognitive Psychology, (104), pp. 57-82

Reviewer #3: This study investigated how people update their actions based on what they learn by observing physical events - in particular, whether new information about a latent variable (mass) is incorporated into behavior in a manner consistent with Newtonian physics. In a clever series of experiments where participants launched pucks attempting to hit a target, the authors found that participants were able to calibrate their launches well in the presence of visual feedback, then carried their assumptions from that experience to their behavior with a new puck of unknown mass, and finally, flexibly updated their behavior on that puck after observing collision events that revealed its mass. Subsequent modeling work showed that the updates to launching behavior were more consistent with Newtonian mechanics than a heuristic model where launch force scales linearly with target distance.

I find the experiments to be well-motivated and well-designed, and the results intriguing, particularly with regard to how readily and precisely participants incorporate their mass inferences into their subsequent behaviors. However, I have a number of concerns about the manuscript in its present form, particularly with regard to the interpretation of the findings as speaking to a link between physical intuitions and actions.

We thank the reviewer for the overall positive evaluation of our manuscript.

1) A primary goal of this study is to investigate how physical intuitions shape actions. I agree that this is an important and understudied question, but I don't feel that the present study makes a convincing case that the results tell us about action per se. Of course participants must always take some kind of action to report on their decisions, but the action here is an arbitrary one that does not invoke the same movements as actually throwing a puck. Eg, participants didn't need to tense their muscles to an appropriate degree or prepare an appropriate grip aperture as in the lifting studies that were cited. The nature of the action may make a substantive difference - engaging the action system in a naturalistic way may affect the way people access their internal physical models (eg, Schwartz & Black, Inferences through imagined actions). Is there any aspect of the study that can establish that the nature of the action was important? Would the results here be different if, say, participants verbalized a number indicating how hard to launch the puck? If not, then I feel the motivation for the paper needs reworking to focus less on the action per se and more on the updating of beliefs, etc.

We fully agree with the reviewer's intuition, that the nature of the action most probably makes a substantive difference. We also agree, that the current manuscript tested whether the physical properties transfer from visual inference to one specific motor planning task and not to all conceivable motor tasks. Transfer to all possible actions or "action per se" is certainly difficult to show and in the end is an empirical question that can be addressed through additional experiments only. This is the reason, why we are careful not to over-generalize our results. Indeed, the title speaks of transfer "to a visuomotor decision task", not all tasks.

Performance of human subjects are known to be notoriously much worse when reasoning and verbalizing continuous probabilistic decisions such as "I would press

the button for the duration of 3,60 seconds compared to actually pressing a button. This has led some authors to recommend converting financial decision making to equivalent visuomotor tasks (Trommershäuser, J., Maloney, L. T., & Landy, M. S. (2008). Decision making, movement planning and statistical decision theory. Trends in cognitive sciences, 12(8), 291-297.).

But it is rather tricky to distinguish different actions particularly in a computational framework. What is an “arbitrary” action and what is a “natural” action in our world? The actions required from subjects in this experiment are continuous and not discrete. Subjects did have to press a button for a certain duration, which arguably is quite natural to our subjects. Lifting a weight would have given subjects an additional sensory cue, which we wanted to exclude. As such, in our view it is particularly remarkable, that subjects were able to learn the required button press-times in as few as 6 trials to reach steady state behavior in phase two of the experiment given visual feedback. Importantly, previous research almost exclusively tested intuitive physical reasoning with forced choice tasks. The current action is instead a continuous button press. In the setting of inferential tasks, especially when modelling all involved uncertainties conveniently as Gaussians, simply picking the value with the highest posterior probability, the MAP, is sufficient. The current study shows, that for an interaction task involving perceptual uncertainty and action variability, the cost function stemming from decision theory needs to be included. Furthermore, because of the known visuomotor variability, the cost function will interact with the perceptual inference process and the press-time variabilities. This is particularly important, as it establishes, that people’s deviations in intuitive physical reasoning can not only be attributed to perceptual uncertainties and prior beliefs but also to the influence of cost functions.

We have revised the paragraph introducing the cost function as follows and hope to have clarified its role:
“However, modeling perception as inference may not be sufficient to describe our participants’ behavior and their selection of actions. Given a posterior over mass and distance describing the perceptual belief of a subject on a particular trial, a specific press-time needs to be selected. In order to model this selection process we take action variability and potential cost functions into account. Cost functions govern which action, here the press-time, should be chosen given a posterior belief and a cost function, which quantifies how the decision process penalizes errors on the task. This means that it is assumed that participants select an action that minimizes potential costs associated with missing the target. Loss functions, describing the rewards or costs for every action in the action space, can have any arbitrary form, nonetheless we chose a set of three standard loss functions and compare their predictions: 0-1, absolute and quadratic loss functions. These three canonical loss functions express subjects’ implicit preferences for reaching a decision about press-times based on a putative perceptual posterior: the 0-1 loss corresponds to penalizing equally all deviations between the chosen value and the correct value, the absolute loss corresponds to penalizing deviations from the true value linearly, and the quadratic loss penalizes the deviations quadratically. It can be shown that these loss functions lead to different decisions for a continuous variable with a non-symmetric distribution [31]. Thus, assuming that humans do have costs for missing the target and associated policies to minimize these costs, leads to three different model versions for each model class (see S1 Appendix, ”Implementation of cost functions”).”

2) Here, the distinction between linear and quadratic distance scaling serves as the diagnostic of whether participants are drawing on a Newtonian model, but it certainly seems possible that participants could adopt quadratic scaling in their launching behaviors without really employing any more sophisticated mental model of physics. Participants may learn the quadratic scaling during the feedback phase and then carry that quadratic scaling through their behavior in the rest of the study as a heuristic that worked well when feedback was available. Are there additional ways in which behaviors based on a full-fledged Newtonian model would differ from one that has quadratic scaling but none of the rest of the sophistication of a Newtonian model? Could any additional distinctions be tested here to further make the case for a Newtonian model?

Thank you for asking this question. This made it clear that we did not do a good job at explaining the results. This question also relates to questions of the other two reviewers. Our answer relates to the fact, that there is not one non-linear function, but there are three non-linear functions, see figure 2.

Let us assume that subjects learned a function for the press times in the phase 2. How could they do that? Because they could not lift an actual puck but only see the different sliding of the red and yellow pucks, there was no explicit cue to mass in any other phase of the experiment than during the collisions. Thus, how much lighter or heavier the red puck was compared to the yellow puck is never revealed. Note also, that no collisions between yellow and red pucks were ever shown. Similarly, the collisions were shown without the influence of friction. If mass is not part of the model, subjects learn two non-linear functions, one for the red puck and one for the yellow puck. Therefore, subjects could not learn one quadratic scaling, if they did not implicitly assume that mass is what distinguishes the red from the yellow puck, that mass needed to be estimated from pucks' dynamics by factoring it out, and then transferring it to the interpretation of the collisions with a new puck.

Thus, if we assume that we learn one scaling for the yellow puck and one scaling for the red puck, how can subjects convert yellow or red to a number that allows converting the two functions into one function? This is only possible under the assumption that the difference between red and yellow pucks is mass. This means, that any sort of transfer of non-linearity is by itself evidence for the application of a Newtonian model.

To make this clearer, we added the following passage in the discussion:
"When visual feedback about the pucks' motion during the feedback phase was available, subjects needed on average only six trials to reach stable performance. This is particularly remarkable, because it corresponds to adjusting the press-times to a single puck's mass over the four initial trials and then adjusting the press-times within only two subsequent trials to a new puck with a different mass. Thus, the observation of the pucks' dynamics over six trials was sufficient to adjust the press-times with the square-root of initial distance, but differently for the two pucks, see Fig. 2. Note that in phase two, subjects only had a contextual color cue distinguishing the two pucks. Therefore, subjects needed to learn two different functions relating the pucks' initial distances to the required press-times, one for each puck. This is a

strong indication, that subjects readily interpreted the differences between red and yellow puck as being caused by the latent masses, which were by itself not observable.

Additionally, we now include the predictions of press-times according to the linear heuristics in the plots “Deviations from fully-observed Newtonian physics and model predictions” in the supplementary. These plots show the large deviations in prescribed press-times between models.

To clarify these points, we have reworked three paragraphs in the discussion, those marked in red in the revised manuscript.

3) For the implicit mass beliefs plotted in Fig. 8 - are the accurate mass beliefs for phase 2 a necessary consequence of the fact that perceptual uncertainty and behavioral variability were estimated for each participant based on the data from that phase? (ie, is there some circularity here?) Or if not - ie, the mass beliefs could have been scattered even though the data was fit in this way - it would be useful to include some intuitive description of how that could have been the case.

We are very thankful to the reviewer for this question. The clear answer to this question is that there is no circularity, but that we did not do a good job at explaining this fact adequately.

First of all, the prior probability of masses is Gamma(6, 2.5). This distribution has 95% of its probability mass between values of m= 0.88 and m= 4.67. So, in principle, the model has 0.95 probability of assigning a mass between 0.88 and 4.67 to the pucks without any data as evidence. The mass belief itself, i.e. the posteriors over masses given the data, determines the gradient of the distance as a function of the press-time. Adding perceptual uncertainty and action variability just further helps to describe the spread of the measured behavior within trials of the same phase. You can think about the mass as a bias/mean of a distribution and about the uncertainties as standard deviation to put it very simple. Taking away the uncertainties will only slightly affect the inferences of the mass but will obviously harm the model in its predictions. Only the cost function can influence the inference of the mass, because of the asymmetric variability in human press-times. Thus, inclusion of perceptual variability and behavioral variability in no way “skewed” inferences of mass beliefs toward the true masses. The space of possible values is actually enlarged.

We described selecting the quadratic cost function on the basis of three analyses: correlations between residual press-times and distances to target, inferred masses, and posterior predictive distributions of the press-times for all models. Based on your question, we make it now clear, that selection of the appropriate cost function is based on the correlations between residual press-times and distances to target, and posterior predictive distributions of the press-times for all models. That the masses under the quadratic cost function are closer to their true value is now report as a result, because this is much more appropriate. Again, we thank the reviewer for this question.

To clarify these points, we have reworked three paragraphs in the discussion, those marked in red in the revised manuscript.

Additionally, we change the text comparing the masses inferred with the different cost function:
“A consequence of selecting the quadratic cost function on the basis of the analyses of press-time residuals and posterior predictive distribution of press-times allows comparing the masses inferred on the basis of participants’ behavior. Remarkably, posterior distributions inferred with data aggregated over participants only from phase two match actual masses implemented in the physical simulations better for the quadratic cost function (see S1 Appendix, ”Latent masses by cost function: aggregated data from phase ’feedback’”).”

minor points:

4) It is hard to know what exactly to take from panel A of fig. 2 - what does the y axis represent? Were there some shots that landed in 4, 5, & beyond that we're not seeing plotted?

Yes, we are very sorry, thank you for catching this! We added axis description: “percentage of shots landed in”. Yes, there were shots in ring 4, 5 and beyond, too.

5) I don't see where Figs. 2 & 3 are referred to in the text of the results. It would be helpful to reference the figures when describing the corresponding results.

We are very sorry, thank you. We have exchanged figures 2 and 3 and we have added the following references to the figures in the text:

“The button press times for all phases of the experiment are shown in Fig. 2.”

“The mean absolute error over the entire phase was 0.928m (0.0177m SEM), see Fig. 3.”

“Mean absolute error was 2.87m (0.104m SEM), see Fig. 3.”

“Performance was significantly higher than in phase three with a mean absolute error of 1.63m (0.0440m SEM), although the puck was the same as in phase three and although subjects did not obtain visual feedback, see Fig. 3.”
